# Characterization of the clonal hierarchy and immunophenotype of *PTPN11* mutations in acute myeloid leukemia

**DOI:** 10.1172/jci.insight.193779

**Published:** 2026-02-23

**Authors:** Sydney Fobare, Chia Sharpe, Kate Quinn, Kinsey Bryant, Linde A. Miles, Robert L. Bowman, Carolyn Cheney, Casie Furby, Marissa Long, Kaytlynn Fyock, Ben Wronowski, James R. Lerma, Krzysztof Mrózek, Deedra Nicolet, Thomas M. Sesterhenn, Megan E. Johnstone, Jianmin Pan, Shesh N. Rai, Chandrashekhar Pasare, Nives Zimmermann, Wen-Mei Yu, Cheng-Kui Qu, Andrew Carroll, Richard Stone, Eunice S. Wang, Jonathan Kolitz, Bayard Powell, John P. Perentesis, Ann-Kathrin Eisfeld, Erin Hertlein, John C. Byrd

**Affiliations:** 1 Medical Scientist Training Program, The Ohio State University, Columbus, Ohio, USA.; 2 Department of Internal Medicine, University of Cincinnati, Cincinnati, Ohio, USA.; 3 Division of Experimental Hematology and Cancer Biology, Cincinnati Children’s Hospital, Cincinnati, Ohio, USA.; 4 Department of Pediatrics, Cincinnati Children’s Hospital Medical Center, Cincinnati, Ohio, USA.; 5 Cancer and Blood Diseases Institute, Cincinnati Children’s Hospital, Cincinnati, Ohio, USA.; 6 Department of Cancer Biology, University of Pennsylvania, Philadelphia, Pennsylvania, USA.; 7 Clara D. Bloomfield Center for Leukemia Outcomes Research, and; 8 Alliance Statistics and Data Management Center, The Ohio State University Comprehensive Cancer Center, Columbus, Ohio, USA.; 9 Biostatistics and Informatics Shared Resource, University of Cincinnati Cancer Center, Cincinnati, Ohio, USA.; 10 Cancer Data Science Center, and; 11 Department of Biostatistics, Health Informatics and Data Sciences, University of Cincinnati College of Medicine, Cincinnati, Ohio, USA.; 12 Pathology and Laboratory Medicine, University of Cincinnati, Cincinnati, Ohio, USA.; 13 Aflac Cancer and Blood Disorders Center, Department of Pediatrics, Winship Cancer Institute, Emory University School of Medicine, Atlanta, Georgia, USA.; 14 Department of Genetics, University of Alabama at Birmingham, Birmingham, Alabama, USA.; 15 Dana-Farber/Partners CancerCare, Boston, Massachusetts, USA.; 16 Roswell Park Comprehensive Cancer Center, Buffalo, New York, USA.; 17 Monter Cancer Center, Zucker School of Medicine at Hofstra/Northwell, Lake Success, New York, USA.; 18 Wake Forest Baptist Comprehensive Cancer Center, Winston-Salem, North Carolina, USA.; 19 Division of Hematology, Department of Internal Medicine, The Ohio State University Comprehensive Cancer Center, Columbus, Ohio, USA.

**Keywords:** Hematology, Oncology, Leukemias, Molecular genetics, Mouse models

## Abstract

Mutations in protein tyrosine phosphatase non-receptor type 11 (*PTPN11*) have been considered late acquired mutations in acute myeloid leukemia (AML) development. Using single-cell DNA sequencing, we found that *PTPN11* mutations can occur as initiating events in some patients with AML when accompanied by strong oncogenic drivers, commonly *NPM1* mutations. The resulting AML has a diverse set of variably differentiated myeloid cells with few myeloid cells that lack leukemic mutations. The role of *Ptpn11* as a codriver was confirmed in a murine model that exhibits an AML phenotype with a comparable immune diversity that is serially engraftable and reconstituted from early precursor cells. Furthermore, lineage-negative bone marrow cells from these mice reconstitute the full diversity of mature myeloid cells, and these cells exhibit an altered cytokine response after physiologic stimulation. Our work highlights how *PTPN11*-mutated AML is derived from a multitude of codominant and late acquired aberrations that have a previously unrecognized differentiated myeloid clonal expansion potentially contributing to pathogenesis of the disease.

## Abstract 2

*PTPN11* mutations in AML can be early events in the clonal evolution of disease development and are associated with variably differentiated myeloid cells.

## Graphical abstract

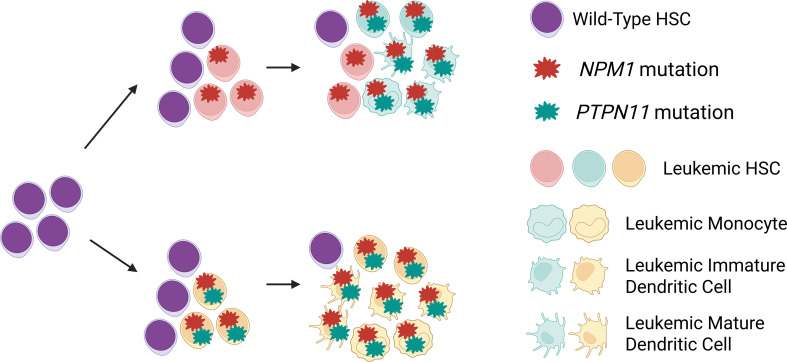

## Introduction

Acute myeloid leukemia (AML) is the most common diagnosed acute leukemia in adults and for most patients it has extremely poor outcomes. In younger patients, leukemogenesis most commonly involves early acquisition of a distinct balanced translocation or mutations with strong oncogenic potential, followed by acquisition of additional late genetic mutations in signaling genes (e.g., *KIT*, *FLT3*, *NRAS*, *KRAS*, *NF1*, *CBL*, and *PTPN11*) (1). In contrast, in most older adults, AML is characterized by the early acquisition of a variety of clonal hematopoiesis (CH) mutations (e.g., *DNMT3A*, *TET2*, and *ASXL1*) that are not by themselves transforming but rather promote inflammation and confer a stem cell/progenitor growth advantage and increased risk of hematologic malignancy and multiple other complications (2). CH mutations are then followed by acquisition of additional signaling mutations or cytogenetic abnormalities that lead to the spectrum of neoplastic development: cytopenias without dysplasia, myelodysplastic syndrome, and/or ultimately AML (3). Characterization of the impact of gene mutations and cytogenetic abnormalities on clinical outcome in AML has been mostly derived from treatment trials with intensive chemotherapy, and as a result a distinct group of driver and late mutations are well characterized as predictors of poor outcome in the 2022 European LeukemiaNet (ELN) classification of AML (4).

The development of now approved targeted agents toward FMS-like tyrosine kinase 3 (FLT3) (midostaurin, gilteritinib, and quizartinib), IDH1 (ivosidenib, olutasidenib), IDH2 (enasidenib), and BCL2 (venetoclax) have transformed the treatment landscape in AML. Within these new targeted therapeutic approaches, many established mutations associated with poor prognosis, such as *TP53* mutations, have continued to predict poor outcomes. However, additional mutations such as MAPK-pathway-activating mutations (e.g., *NRAS*, *KRAS*, *CBL*, *NF1*, and *PTPN11*) have emerged as specifically impacting the outcomes of patients receiving targeted therapies (5–8). Indeed, patients with *PTPN11*-mutated AML had the lowest response among patients carrying other genomic abnormalities who received IDH2 inhibitors (9) or venetoclax/azacitidine (10, 11). Studies to identify the pathogenic features of clonal evolution and compound genetic models of *PTPN11*-mutated AML are lacking.

The *PTPN11* gene encodes Src homology region 2–containing (SH2-containing) protein tyrosine phosphatase 2 (SHP2). SHP2 contains 2 SH2 domains and a tyrosine phosphatase domain. SHP2 is capable of self-regulation, as the N-terminal SH2 domain can bind to the active phosphatase domain to prevent dephosphorylation of SHP2 targets (12). Pathogenic *PTPN11* mutations tend to occur in regions involved in its self-regulation, and thus, these gain-of-function mutations result in activation of numerous signaling pathways (e.g., RAS/ERK1/2, FLT3, JAK/STAT, PI3K/AKT, and NF-κB), mediates immune evasion via PD-1, and induces the overexpression of antiapoptotic proteins such as MCL-1 (13–15). In humans, gain-of-function *PTPN11* mutations can occur as germline mutations in Noonan syndrome and are associated with a higher frequency of juvenile myelomonocytic leukemia, suggesting these mutations can contribute to the initiation of myeloid malignancies (16, 17). To study the role of *PTPN11* gain-of-function mutations, Xu et al. developed the *Ptpn11^E76K^* mouse that mimics the most common hotspot mutation in both Noonan syndrome and myeloid malignancies (18). They found that constitutive expression of *Ptpn11^E76K^* is embryonically lethal in mice, but conditional, heterozygous expression of the *Ptpn11^E76K^* mutation under the control of Mx-Cre promotes the development of multilineage leukemias (approximately equal frequency of myeloid, B cell, and T cell leukemias).

These findings have prompted several groups, including our own, to examine the prognostic impact of *PTPN11* mutations in AML. These analyses suggest that *PTPN11* mutations co-occur most commonly with either *NPM1* mutations or with chromosome translocations or inversions and typically confer poor outcome (19–21). In the absence of an *NPM1* mutation, *PTPN11* mutations often co-occur with cytogenetic abnormalities associated with dysregulation of *EVI1*. Inconsistencies between studies suggest that the pathogenic contribution of *PTPN11* mutations may depend on its context among other driver mutations (19). Notably, in some patients, *PTPN11* mutations occur at a high variant allele frequency (VAF), suggesting they could be initiating or co-initiating events in leukemogenesis. However, due to limitations of previous sequencing technologies it has not been possible to determine when *PTPN11* mutations arise during AML development. Understanding the clonal evolution of *PTPN11* mutations is clinically relevant, as previous reports have suggested that in patients treated with intensive chemotherapy, dominant *PTPN11* mutations are associated with better outcomes compared with subclonal mutations, although this is confounded by the fact that patients with subclonal mutations tend to have a higher comutational burden (20). However, as an early driver mutation *PTPN11* has serious implications for predicting therapeutic response, and as an acquired subclonal mutation *PTPN11* can contribute to the emergence of resistance to therapy, both resulting in poor outcome. Given the lack of knowledge related to clonal evolution of *PTPN11* mutations and characterization of myeloid immunophenotype of this disease, we initiated studies in both primary human samples and developed what we believe is the first murine model of concurrent *Npm1* and *Ptpn11* mutations to address these questions. Herein, we demonstrate that *PTPN11* can be an early acquired mutation, commonly co-occurring with a strong oncogene (*NPM1* mutation) to promote AML development. The *NPM1*- and *PTPN11*-mutated subtype of AML has a diverse immune profile in patients that was reproduced in our *Npm1^cA^/Ptpn11^E76K^* animal model that exhibits capacity for serial engraftment. This model provides a system to understand the role of early *PTPN11* mutations as they relate to clonal architecture and influence on AML immunophenotype.

## Results

### PTPN11-mutated AML demonstrates multiple-ancestor origins.

**Figure 1:**
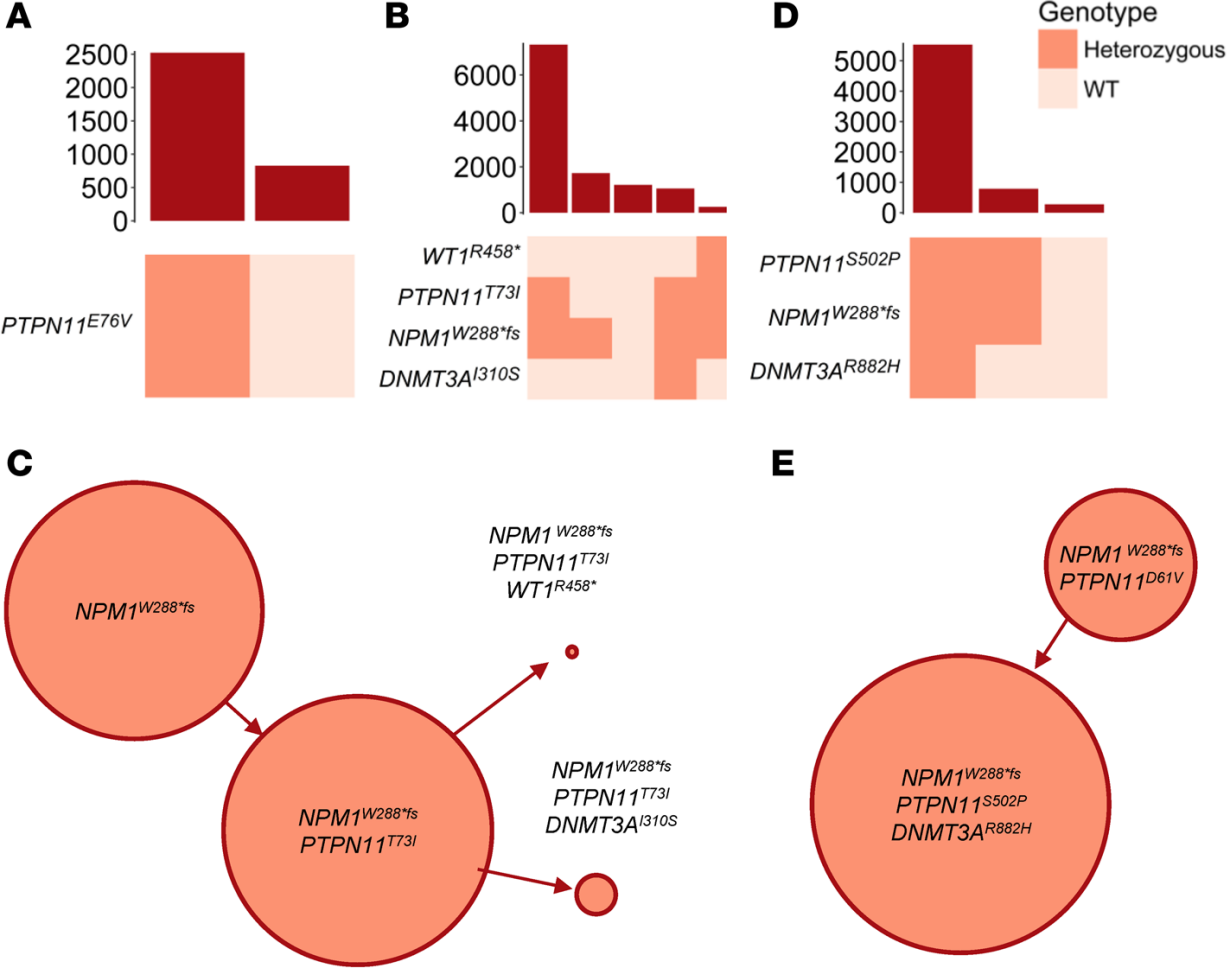
Clonal architecture of *PTPN11*-mutated AML patient samples. Clonographs demonstrating the frequency of co-occurring mutations determined using scDNA-seq in representative samples. (**A**) *PTPN11* mutation without *NPM1* mutation (AML-3; total *n* = 4). (**B**) Subclonal *PTPN11* mutation (AML-16, total *n* = 4). (**C**) Inferred clonal relationship for **B**. (**D**) Early *PTPN11* mutation (AML-9, total *n* = 5). (**E**) Inferred clonal relationship for **D**.

Previous work from our group demonstrated that *PTPN11* mutations occur at a wide range of VAFs (0.05 up to 0.54), suggesting that *PTPN11* mutations may occur early in leukemogenesis (19). Previous studies that have evaluated *PTPN11* mutations using single-cell sequencing techniques have mainly included low-frequency *PTPN11* mutations (22–24). To further elucidate the genomic characteristics of dominant *PTPN11* mutations, we assembled a cohort of patients enriched with those that had a VAF of greater than 0.3 and mutations that fell within the *PTPN11* amplicons generated by the Tapestri single-cell DNA sequencing (scDNA-seq) Myeloid Clonal Evolution Published Panel (Mission Bio). All Alliance patient samples that we had access to and met these 2 requirements were included in the study. Four patients with confirmed *PTPN11* mutations without a co-occurring *NPM1* mutation by bulk sequencing and 9 with concurrent *PTPN11* and *NPM1* mutations were analyzed. The demographics and mutation profile identified by bulk sequencing (VAF > 0.05) of the included patients are summarized in Table 1. All 4 patients without *NPM1* mutations had a strong driver translocation or inversion involving *EVI1* together with monosomy 7 (cases AML-1 to AML-3) or t(6;11) (q27;q23) (case AML-5). However, chromosomal changes are not captured by this scDNA-seq panel, so in these samples a single *PTPN11*-mutated clone was identified (representative case AML-3; Figure 1A and Supplemental Figure 1; supplemental material available online with this article; https://doi.org/10.1172/jci.insight.193779DS1).

Among the 9 patients with both *PTPN11* and *NPM1* mutations, we identified that half the patients had subclonal *PTPN11* mutations and the other half had *PTPN11* mutations in the first leukemic clone identified. Two patient samples (AML-11 and AML-16) showed evidence of an *NPM1*-mutated clone that lacked a *PTPN11* mutation (representative case AML-16; Figure 1B and Supplemental Figure 1), suggesting that the *NPM1* mutation preceded the *PTPN11* mutation. However, the double-mutant cells still generated the dominant clone (Figure 1C). Two patient samples (AML-13 and AML-15) had the acquisition of a *TET2* or *DNMT3A* mutation followed by *PTPN11* and then *NPM1* mutations. The clonal architecture in these 4 samples aligns with the predominant understanding of *PTPN11* mutations as a late acquired signaling mutation akin to *FLT3* or *RAS* family mutations and is supported by previous reports of scDNA-seq performed on samples from patients with AML (22, 23). In the remaining 5 patients (AML 6–10), we observed *PTPN11* mutations in the earliest detectable clone (representative case AML-9; Figure 1D and Supplemental Figure 1). In 4 of these 5 patients, no single-mutant clone was identified, which precluded the ability to confirm the initiating mutation in these samples. Nevertheless, these data support the early emergence of a *PTPN11* mutation in the clonal evolution of these patients (Figure 1E). These data align with previous single-cell sequencing studies where signaling mutations can occur prior to *NPM1* mutations (24). In 1 patient sample, we identified a *PTPN11* single-mutant clone in addition to a *PTPN11* and *DNMT3A* double-mutant clone and a *PTPN11*, *DNMT3A*, and *NPM1* triple-mutant clone. This suggests that in some patients *PTPN11* mutations may be an early event in AML leukemogenesis and can occur prior to the acquisition of other mutations typically described as driver mutations (AML-8; Supplemental Figure 1). Collectively, these data support the conclusion that while *PTPN11* mutations are frequently a late acquired event, in contrast to other *RAS* family mutations, they can also serve as a driver or codriver mutations in AML when coupled with mutations or chromosomal abnormalities that are strongly associated with myeloid oncogenesis.

### Most myeloid cells in PTPN11-mutated AML have leukemic mutations at diagnosis.

**Figure 2:**
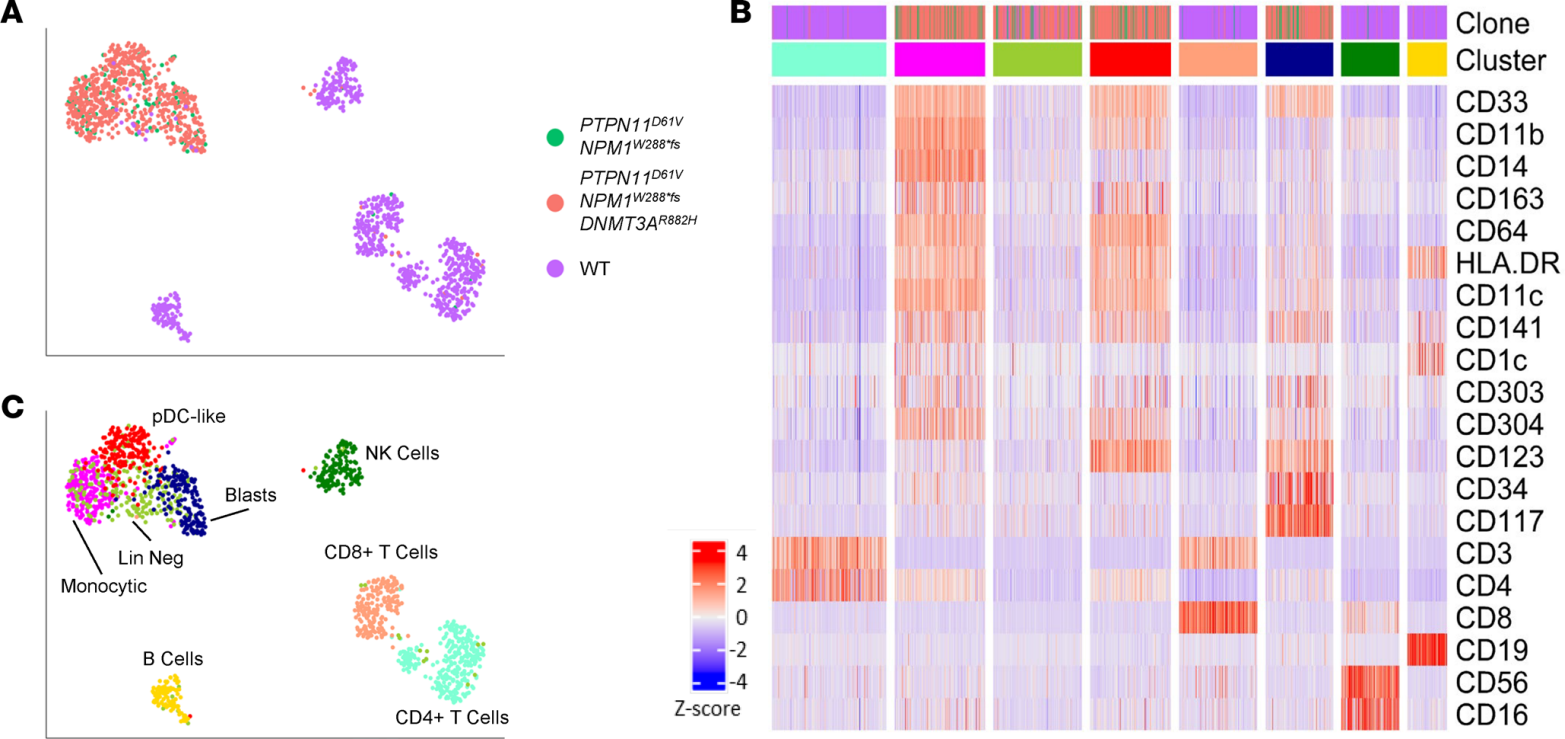
Mutated AML cells comprise most myeloid cells at diagnosis. Immunophenotypic clusters were identified using shared nearest neighbor clustering on a representative early *PTPN11*-mutated sample (AML-6). (**A**) UMAP overlaid with WT, *NPM1*^287^/*PTPN11*^D61V^, and *NPM2*^288*fs^/*PTPN11*^D61V^/*DNMT3A*^R882H^ clone identity and nearest neighbor clustering. (**B**) Heatmap of select lineage defining markers. A total of 9 samples were analyzed. (**C**) UMAP overlaid with the identity of the clusters from the heatmap in **B**.

Concurrent with the scDNA-seq results (Figure 1), we performed simultaneous single-cell immunophenotyping in primary AML samples using an antibody panel against 42 antigens (Mission Bio Tapestri). Distinct immunophenotypic clusters were identified using shared nearest neighbor clustering. We first generated a uniform manifold approximation and projection (UMAP) for data reduction based on the surface marker expression. As expected, the AML clones clustered separately from the wild-type (WT) cells within a patient sample (AML-6; Figure 2A). Heatmaps were also generated to visualize the coexpression of cell surface markers. Here we can identify clusters of lymphoid cells without leukemic mutations (yellow, teal, tan, and dark green clusters). Within the clusters associated with leukemic mutations, there are cells that express very few myeloid markers (light green cluster), immature myeloid markers such as CD117, CD34, and CD123 (navy cluster), and clusters that express mature myeloid markers such as CD14, CD11b, and HLA-DR (pink cluster) or CD11c and CD141 (red cluster, Figure 2B). However, we could not identify a cluster with mature myeloid markers composed of predominately WT cells, thus verifying that the mature myeloid cells in these patients are leukemia derived (Figure 2C). This trend was consistent across all 13 samples analyzed (Supplemental Figure 2). Therefore, our data suggest that AML patients with *PTPN11* mutations have a diverse immunophenotype, with leukemic cells expressing a variety of mature myeloid markers and very few WT myeloid cells at diagnosis.

### The diverse immunophenotype of PTPN11-mutated AML is recapitulated with flow cytometry.

**Figure 3:**
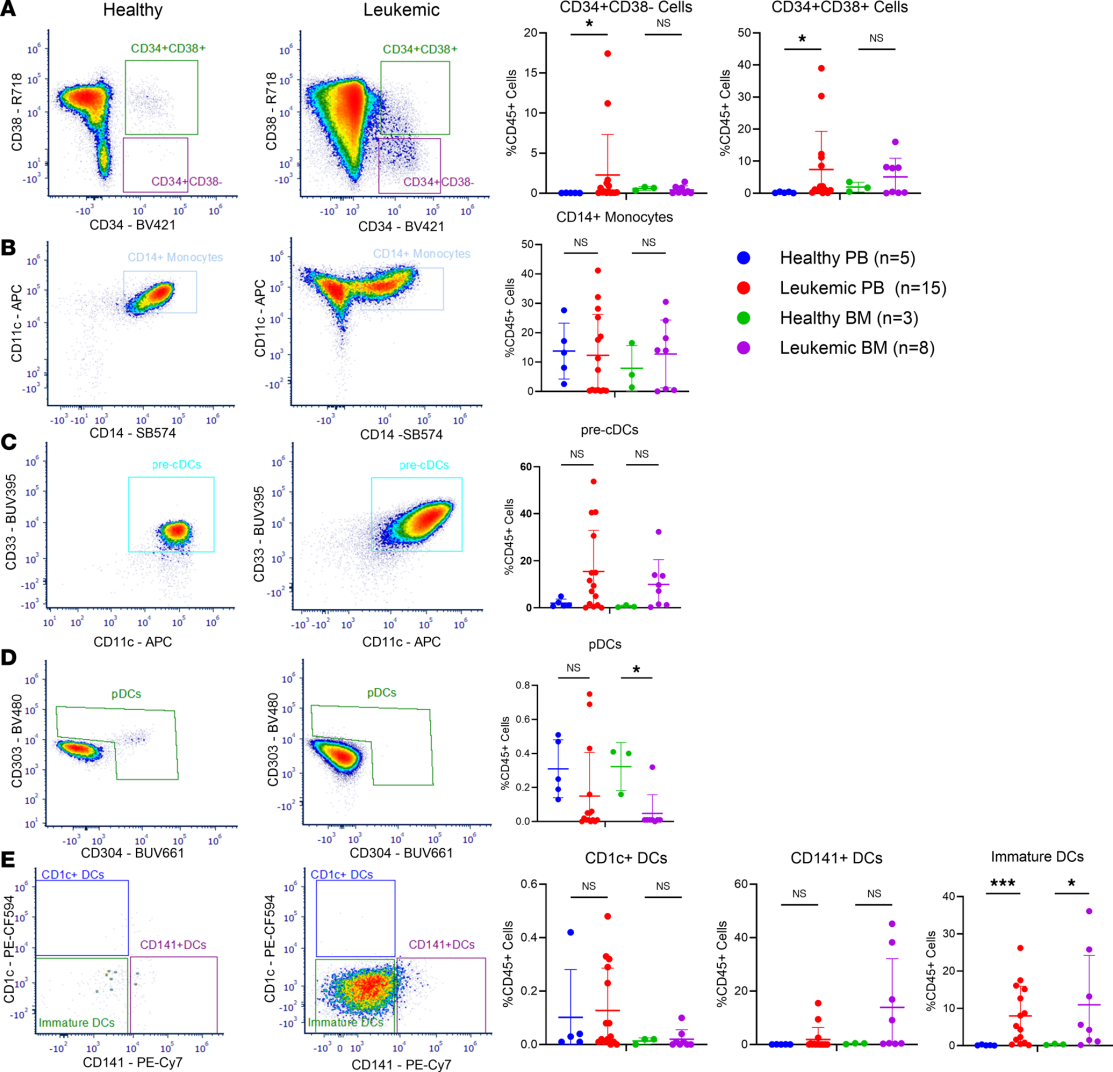
Immunophenotypic diversity of primary *PTPN11*- and *NPM1*-mutated AML peripheral blood and bone marrow samples at diagnosis. (**A**) Percentage of CD34^+^CD38^–^ and CD34^+^CD38^+^ cells in healthy and leukemic peripheral blood (PB) and bone marrow (BM) with representative flow plots. (**B**) Percentage of CD14^+^ monocytes in healthy and leukemic PB and BM with representative flow plots. (**C**) Percentage of pre-conventional dendritic cells (pre-cDCs) in healthy and leukemic PB and BM with representative flow plots. (**D**) Percentage of plasmacytoid DCs (pDCs) in healthy and leukemic PB and BM with representative flow plots. (**E**) Percentage of CD1c^+^ DCs, CD141^+^ DCs, and immature DCs in healthy and leukemic PB and BM, respectively, with representative flow plots. Percentages are based on CD45^+^ cells. Data are presented as mean ± SD of 2 independent experiments. Statistical analysis by 1-way ANOVA with Benjamini-Hochberg FDR correction applied. *FDR-adjusted *P* ≤ 0.05, ***FDR-adjusted *P* ≤ 0.001.

To further validate the diverse immunophenotype of the AML cells as suggested by the single-cell immunophenotype, we developed a spectral flow cytometry immunophenotyping antibody panel against 38 antigens (gating strategy in Supplemental Figure 3). AML leukemic stem cells are canonically defined as CD34^+^ cells (25), but interestingly, we did not identify a large portion of CD34^+^ cells within the *PTPN11*-mutated samples (Figure 3A). This is consistent with previous data showing that *NPM1* mutations are associated with decreased CD34 expression (26). We did, however, note the presence of cells committed to a mature myeloid lineage. In our cohort, we found that about half the patients had an expansion of CD14^+^ monocytes above the mean; however, this was not statistically significant (Figure 3B). There was also a trend toward an increase in pre-conventional dendritic cells (pre-cDCs) in bone marrow of patients with *PTPN11* mutations at diagnosis (FDR-adjusted *P* = 0.0781; Figure 3C). There were a couple of patients with increased plasmacytoid DCs (pDCs) in the peripheral blood but not sufficient to be characterized as pDC-AML (Figure 3D). There was also an expansion of immature DCs in both the peripheral blood and bone marrow compared with healthy controls (FDR-adjusted *P* = 0.0004 and FDR-adjusted *P* = 0.0681, respectively; Figure 3E). Overall, we found that the tumor-derived myeloid cells exhibit a range of early differentiated cells predominantly of monocytic and DC lineages. When these cells are stimulated with CpG overnight, they have a reduced capacity to produce IFNs compared with healthy controls, highlighting a functional difference from normal, nonmutant cells (Supplemental Figure 4). Therefore, the combination of scDNA-seq and flow cytometry data highlights the presence of mutated leukemia cells with a variable immune phenotype representing both immature and mature populations.

### Npm1^cA^/Ptpn11^E76K^ mice consistently develop AML.

**Figure 4:**
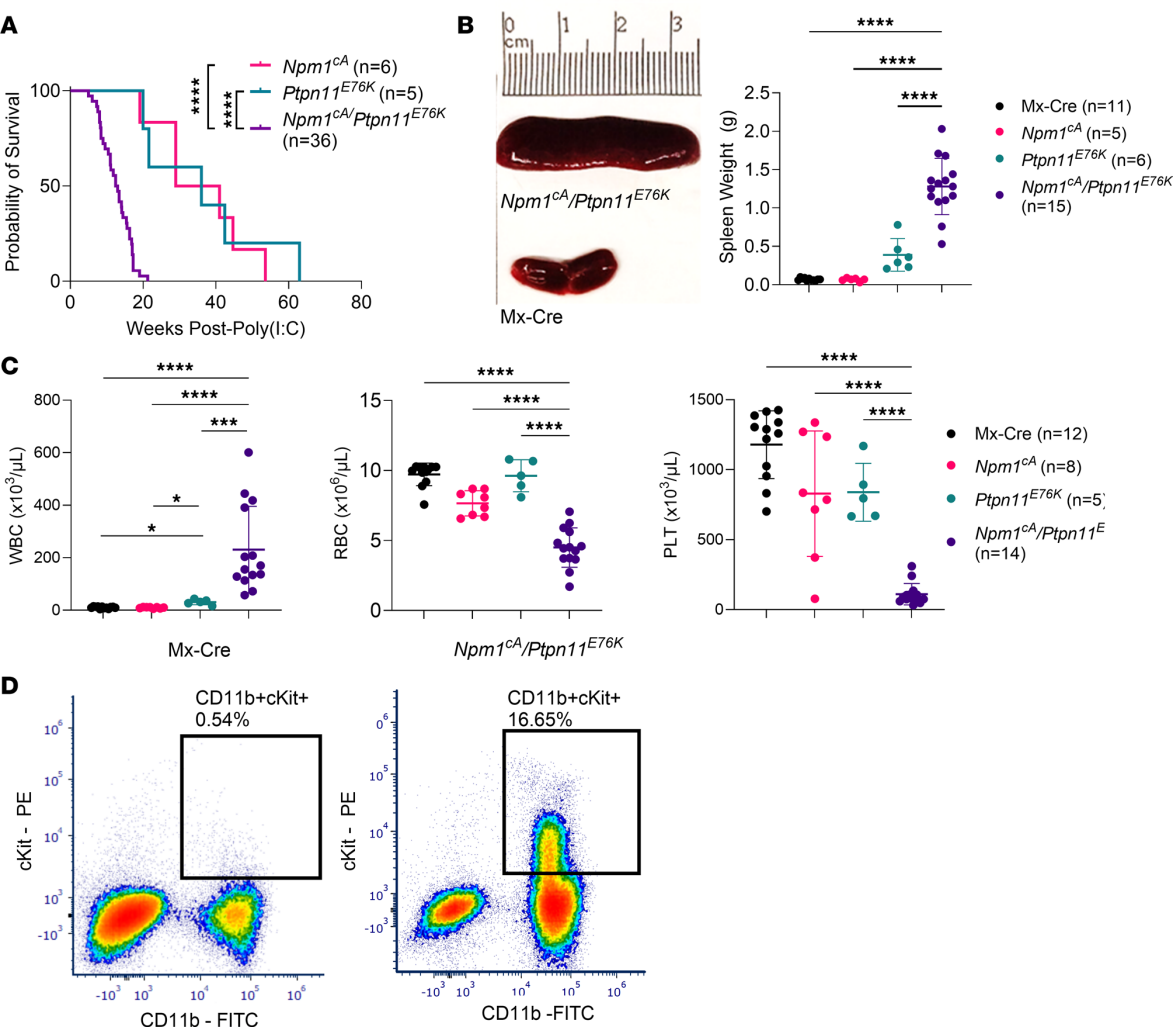
Characterization of the *Npm1*^cA^/*Ptpn11*^E76K^ mouse model. (**A**) Overall survival of *Npm1*^cA^, *Ptpn11*^E76K^, and *Npm1*^cA^/*Ptpn11*^E76K^ mice. (**B**) Representative picture of a spleen collected from a *Npm1*^cA^/*Ptpn11*^E76K^ mouse at survival endpoint compared with age-matched Mx-Cre mouse spleen. Summary of spleen weights (in grams) of *Npm1*^cA^/*Ptpn11*^E76K^ mice at time of death compared with aged-matched controls. (**C**) White blood cell (WBC), red blood cell (RBC), and platelet (PLT) counts of *Npm1*^cA^/*Ptpn11*^E76K^ mice at time of death compared with aged-matched controls. (**D**) Representative peripheral blood flow cytometry plot (gated on CD45^+^CD3^–^CD19^–^NK1.1^–^ cells; percentage based on total CD45^+^ cells) of Mx-Cre and *Npm1*^cA^/*Ptpn11*^E76K^ mice 4 weeks after poly(I:C) induction (total *n* of 11 Mx-Cre mice and 14 *Ptpn11*^E76K^/*Npm1*^cA^ mice). Data are presented as mean ± SD. Statistical analysis by 1-way ANOVA with Benjamini-Hochberg FDR correction applied. *FDR-adjusted *P* ≤ 0.05, ***FDR-adjusted *P* ≤ 0.001, ****FDR-adjusted *P* ≤ 0.0001.

Our AML patient data suggest that *PTPN11* mutations can constitute a driving transformative event and can produce a diverse myeloid phenotype. Therefore, we aimed to develop a *Ptpn11*-mutated AML mouse model that would allow further investigation of the role of *Ptpn11* mutations in leukemogenesis and pathogenesis. Analysis of co-occurring mutations with *PTPN11* aberrations demonstrated *NPM1* to be most co-associated in AML. We therefore combined *Ptpn11*^E76K^ (18) and *Npm1*^cA^ (27) mutant alleles, which are both driven by Mx-Cre (28) and are inducible with IFN or polyinosinic-polycytidylic acid [poly(I:C)]. Mice expressing both mutant alleles (*Npm1*^cA^/*Ptpn11*^E76K^) exhibited a fully penetrant lethal disease phenotype with a median overall survival of 13.4 weeks after induction, significantly faster than either *Ptpn11*^E76K^/Mx-Cre (*Ptpn11*^E76K^; 39.2 weeks) or *Npm1*^cA^/Mx-Cre (*Npm1*^cA^; 35 weeks) mice (Figure 4A). When comparing overall survival of *Npm1*^cA^/*Ptpn11*^E76K^ mice based on sex, female mice had a significantly shorter survival compared with male mice (Supplemental Figure 5).

At time of sacrifice, *Npm1*^cA^/*Ptpn11*^E76K^ mice had significant splenomegaly compared with age-matched Mx-Cre and single-gene control mice (Figure 4B). *Npm1*^cA^/*Ptpn11*^E76K^ mice also exhibited significant leukocytosis, anemia, and thrombocytopenia compared with age-matched single-gene controls (Figure 4C). There was no difference in splenomegaly, leukocytosis, anemia, or thrombocytopenia between sexes at time of death (Supplemental Figure 5). As previously published, the *Ptpn11*^E76K^ mouse can develop multilineage leukemias; therefore, we wanted to confirm that the addition of an *Npm1*^cA^ mutation would result in a consistent myeloid phenotype. We performed flow cytometric analysis, showing that all *Npm1*^cA^/*Ptpn11*^E76K^ mice have a clear expansion of CD11b^+^c-Kit^+^ cells in the blood as early as 4 weeks after induction, which progressively expanded throughout disease development (Figure 4D).

### Broad immunophenotyping identifies diverse myeloid populations in the Npm1^cA^/Ptpn11^E76K^ mice reflective of human AML.

**Figure 5:**
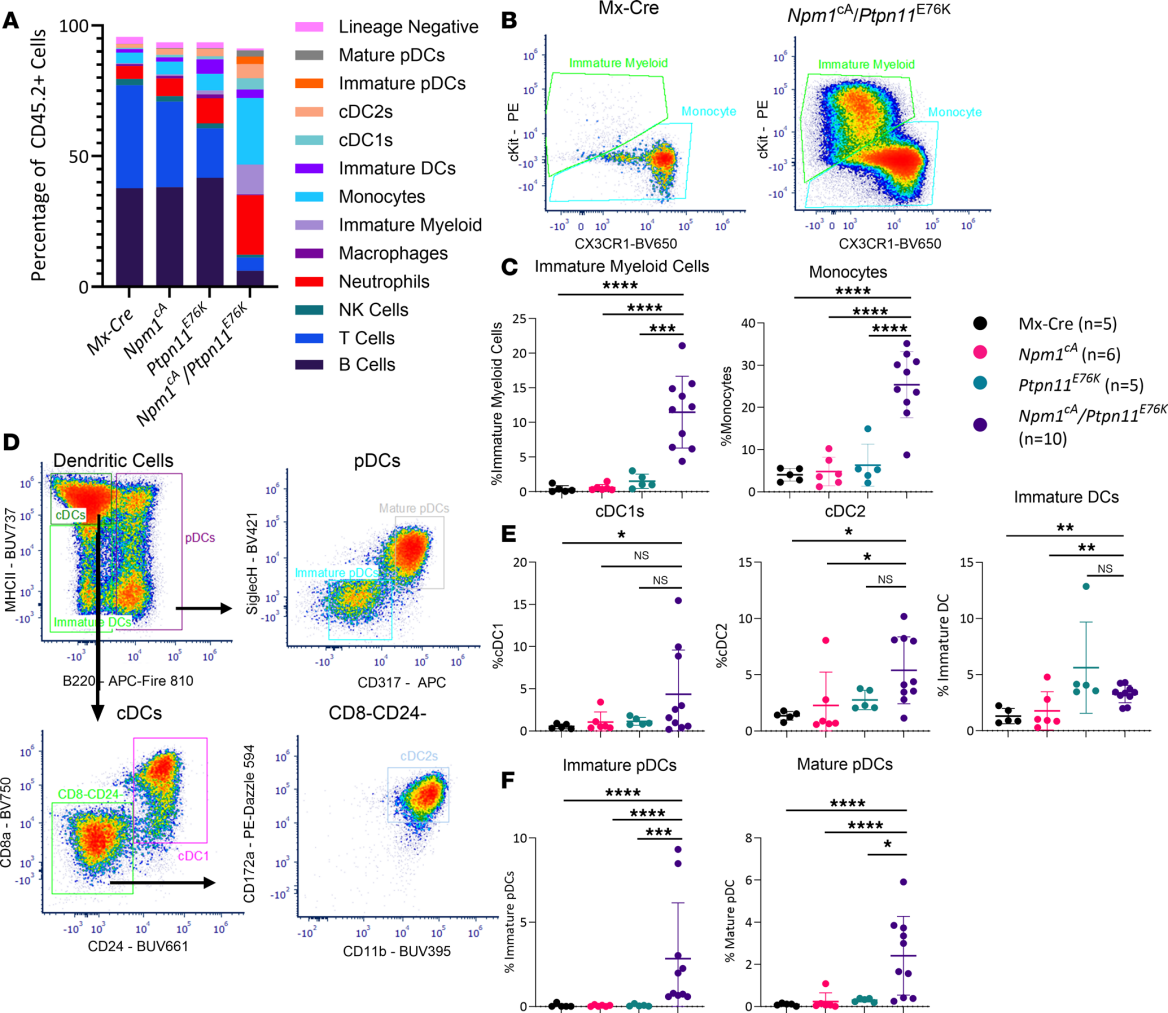
Immunophenotyping of *Npm1*^cA^/*Ptpn11*^E76K^ splenocytes. Flow cytometric analysis of splenocytes from Mx-Cre (*n* = 5), *Npm1*^cA^ (*n* = 6), *Ptpn11*^E76K^ (*n* = 5), and *Npm1*^cA^/*Ptpn11*^E76K^ (*n* = 10) mice. Percentages were calculated as a fraction of total CD45.2^+^ cells and all control mice were age matched. (**A**) Percentage of immune subsets in the spleen. (**B**) Representative flow plots CX3CR1 and c-Kit expression on myeloid cells in a representative Mx-Cre and *Npm1*^cA^/*Ptpn11*^E76K^ mouse. (**C**) Percentage of immature myeloid cells and monocytes. (**D**) Representative flow plots of dendritic cell (DC) gating from a *Npm1*^cA^/*Ptpn11*^E76K^ mouse. (**E**) Percentages of conventional DC 1 (cDC1), cDC2, and immature DC populations. (**F**) Percentages of immature and mature plasmacytoid DC (pDC) populations. Data are presented as mean ± SD from 3 independent experiments. Statistical analysis by 1-way ANOVA with Benjamini-Hochberg FDR correction applied. *FDR-adjusted *P* ≤ 0.05, **FDR-adjusted *P* ≤ 0.01, ***FDR-adjusted *P* ≤ 0.001, ****FDR-adjusted *P* ≤ 0.0001. NK cells, natural killer cells.

As we noted a diverse immunophenotype in primary human AML samples at diagnosis, we comprehensively characterized the *Npm1*^cA^/*Ptpn11*^E76K^ murine disease immunophenotype to see how well this represented human disease. To this end, we developed a spectral flow cytometry immunophenotyping antibody panel against 35 antigens to characterize both the lymphoid and myeloid populations in the spleen (gating strategy in Supplemental Figure 6). Compared with age-matched single-gene controls, the *Npm1*^cA^/*Ptpn11*^E76K^ mice exhibited an expansion of multiple distinct myeloid populations, including neutrophils (CD11b^+^Ly6G^+^) and monocytic cells (CD11b^+^Ly6C^+^) (Figure 5A). As *PTPN11*-mutated AML is associated with a monocytic phenotype (21), it was unsurprising to find that the *Npm1*^cA^/*Ptpn11*^E76K^ mice have an increase in the percentage of monocytes compared with control mice. However, in addition to the expected expansion of the CX3CR1^–^c-Kit^+^ population of “immature” monocytic cells, we also observed a significant expansion of the CX3CR1^+^c-Kit^–^ population of “mature” monocytes (Figure 5, B and C). This suggests that while *Npm1* mutations have been associated with differentiation block, in the context of a co-occurring *Ptpn11* mutation, there is an expansion of variably differentiated myeloid cells, including those immunophenotypically consistent with mature monocytes.

In addition to the expansion of monocytes, we also saw an expansion of variably differentiated DCs in the murine splenocytes comparable to the primary AML samples (Figure 3). Specifically, the *Npm1*^cA^/*Ptpn11*^E76K^ mice have an expansion of cDC1s and cDC2s in the spleen. There was also an expansion of immature DCs (CD11c^+^MHC^lo/–^) compared with Mx-Cre and *Npm1*^cA^ control mice but not to the *Ptpn11*^E76K^ mice, suggesting a relationship to the *Ptpn11* mutation (Figure 5, D and E). There was also an increased percentage of immature (CD11c^+^B220^+^) and mature pDCs (CD11c^+^B220^+^Siglec H^+^CD317^+^) in the *Npm1*^cA^/*Ptpn11*^E76K^ mice compared with controls (Figure 5F). There was no difference in the immunophenotype of the splenocytes from the male and female *Npm1*^cA^/*Ptpn11*^E76K^ mice, suggesting that the difference in survival is not related to an expansion of a specific myeloid cell (data not shown). Therefore, our *Npm1*^cA^/*Ptpn11*^E76K^ mouse can recapitulate the monocytic and cDC expansion of human *PTPN11*-mutated AML and also exhibits an expansion of pDCs.

### Npm1^cA^/Ptpn11^E76K^ splenocytes can recapitulate a leukemic phenotype in primary and secondary recipients.

**Figure 6:**
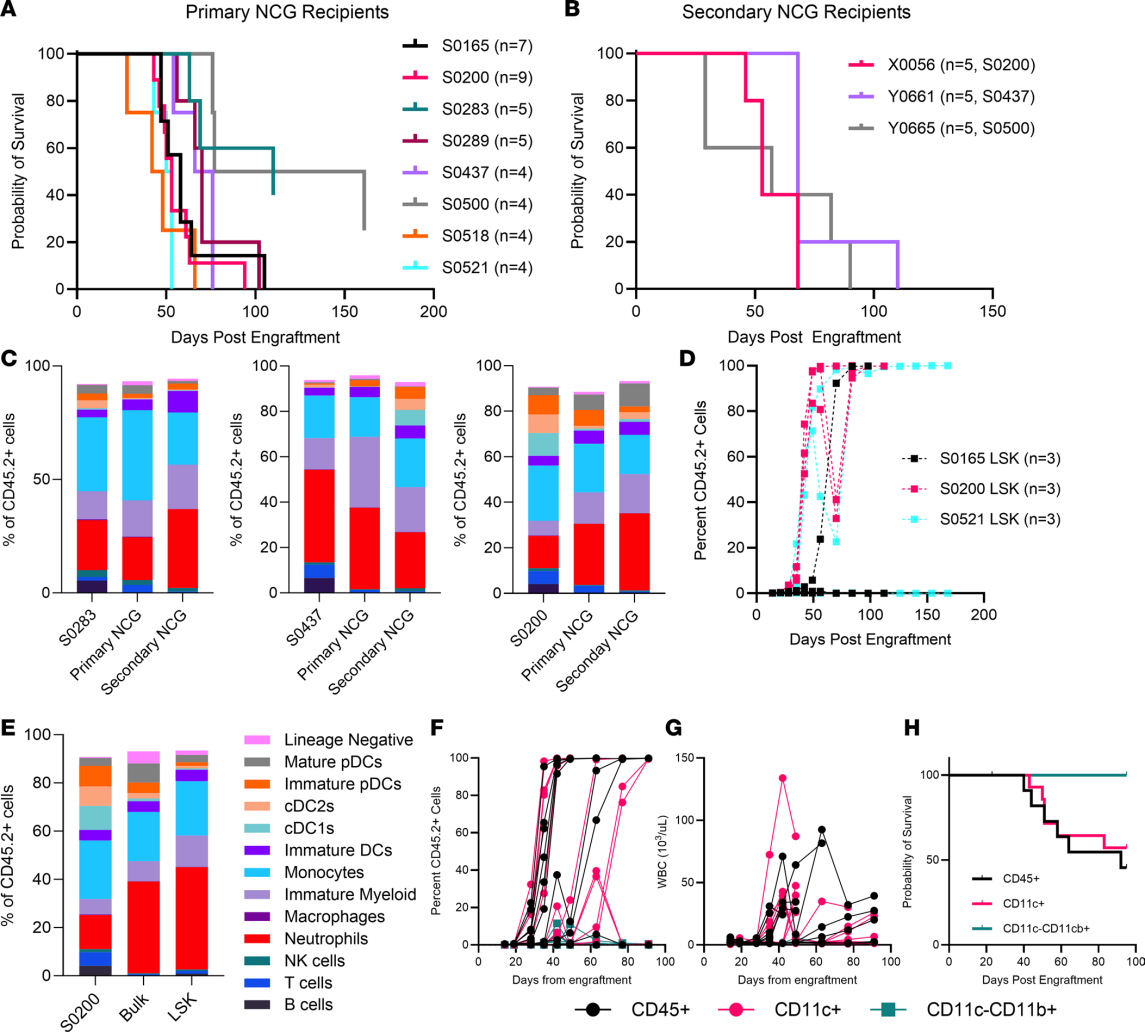
Engraftment of *Npm1*^cA^/*Ptpn11*^E76K^ splenocytes into immunodeficient mice. (**A**) Overall survival in primary NCG engraftment in 3 independent experiments. (**B**) Overall survival in secondary NCG engraftment. (**C**) Summary of spleen immune subsets as a proportion of total CD45.2^+^ cells in primary AML donors compared to primary and secondary NCG recipients meeting the survival endpoint from 2 independent experiments. (**D**) Frequency of donor-derived CD45.2^+^ cells over time in the peripheral blood from NCG mice engrafted with LSK cells. (**E**) Immune cell subsets in spleen from 1 donor (S0200) and recipients of either bulk splenocytes (*n* = 4 recipients) or LSK cells (*n* = 3 recipients) at endpoint. FACS-isolated CD45^+^, CD11c^+^, or CD11c^–^CD11b^+^ cells were sorted from 3 donors. (**F**) Frequency of donor-derived CD45.2^+^ cells over time in the peripheral blood. (**G**) White blood cell (WBC) and (**H**) overall survival of NCG mice engrafted with CD45^+^ (*n* = 12), CD11c^+^ (*n* = 11), or CD11c^–^CD11b^+^ (*n* = 8) cells. Data are presented as the mean. pDCs, plasmacytoid dendritic cells; cDC2s, conventional dendritic cells 2; cDC1s, conventional dendritic cells 1; DCs, dendritic cells; NK cells, natural killer cells.

Thus far, we have demonstrated that co-induction of *Npm1*^cA^ and *Ptpn11*^E76K^ in mice is comparable to our human data and that these 2 mutations are sufficient to develop a 100% penetrant, aggressive AML. However, a hallmark of a true AML model requires that it can be adoptively transferred with preservation of its immunophenotypic features. We therefore wanted to determine whether AML cells from the *Npm1*^cA^/*Ptpn11*^E76K^ mice formed a lethal leukemia in a primary recipient. We found that these splenocytes, when injected via the tail vein of NCG recipients, were able to recapitulate a leukemic phenotype with an average overall survival of 8.2 weeks after engraftment (individual donors [*n* = 8] ranged from 7.5 to 17 weeks, Figure 6A). While all individual AML donors formed a lethal leukemia in some recipients, 2 of the 8 donors tested (S0283 and S0500) had slightly more inconsistent engraftment, resulting in failed engraftment in 2 of 5 and 1 of 4 recipients, respectively. We then took passaged splenocytes from 3 donors (S0200, S0437, and S0500) and engrafted them into additional recipients (*n* = 5 recipients per donor) and found they were capable of engrafting into a secondary recipient with an average overall survival of 8.5 weeks after engraftment (individual donors [*n* = 3] ranged from 7.6 to 9.7 weeks, Figure 6B). The consistency increased with the secondary engraftment, with all 5 recipients from each donor resulting in a lethal leukemia. To confirm that the leukemic cells developing in the recipients phenotypically matched the original donor cells, we performed the immunophenotyping panel on splenocytes from mice with overt leukemia, as described above. We found the overall diversity of cell subsets shows a similar profile to that of the donor in both the primary and secondary engraftment. The variability of differentiated monocytes and DCs was maintained at similar proportions in both the primary and secondary recipients (Figure 6C), suggesting stability of the diverse immunophenotype of *Npm1*^cA^/*Ptpn11*^E76K^ leukemia cells.

### Npm1^cA^/Ptpn11^E76K^ LSKs can recapitulate the immune subset diversity in primary recipients.

With Mx-Cre–driven expression, all hematopoietic lineage cells in the *Npm1^cA^/Ptpn11^E76K^* mice express the driver mutations. However, not all leukemia populations are expected to have the same capacity to engraft into primary recipients. Given the relative stability of the immune diversity of the *Npm1*^cA^/*Ptpn11*^E76K^ leukemic cells, we wanted to determine whether lineage^–^Sca-1^+^cKit^+^ (LSK) cells, which previous studies in mice have identified as containing leukemia-initiating cells (29), were capable of generating diversity of immune subsets identified in the transgenic mice. Therefore, LSKs were sorted from the spleens of individual donors (*n* = 3) and injected via the tail vein into NOD-*Prkdc^em26Cd52^Il2rg^em26Cd22^*/NjuCrl (NCG) recipients (*n* = 3; sorting strategy in Supplemental Figure 7). There was considerable variability in the engraftment of the LSK cells into NCG recipients, with donors producing a lethal leukemia in 1 of 3, 2 of 3, and 3 of 3 recipients (Figure 6D). We again performed immune profiling of the splenocytes from recipient mice that developed a lethal leukemia, and the immune subset diversity in LSK-engrafted mice was comparable to bulk-engrafted recipients (Figure 6E). This suggests that the diverse mature myeloid populations in *Npm1*^cA^/*Ptpn11*^E76K^ AML are derived from a stem or progenitor population within the LSK cells.

### CD11c^+^ Npm1^cA^/Ptpn11^E76K^ splenocytes form a lethal leukemia in primary recipients.

We observed that the rate of engraftment failure in recipients of sorted LSK cells was higher than when bulk splenocytes were engrafted. We therefore hypothesized that a population expressing a lineage marker may also contribute to engraftment. To test this, we sorted 3 populations: (a) total CD45^+^, (b) CD11c^+^ cells (DC lineage) and (c) CD11c^–^CD11b^+^ (monocyte/macrophage lineage) from *Npm1*^cA^/*Ptpn11*^E76K^ splenocytes (*n* = 3; sorting strategy in Supplemental Figure 8). The frequency of donor-derived (CD45.2^+^) engraftment in NCG recipients shows that both the CD45^+^ and CD11c^+^ populations engrafted at an equivalent rate, while the CD11c^–^CD11b^+^ population exhibited highly sporadic and short-lived engraftment (Figure 6F). Both the CD45^+^ and CD11c^+^ populations, but not CD11c^–^CD11b^+^ cells, exhibited increased white blood cell (WBC) counts (Figure 6G), leading to the formation of a lethal leukemia (Figure 6H). Importantly, the survival in mice engrafted with the CD11c^+^ population was similar to NCG mice engrafted with total bulk spleen cells (as in Figure 6A).

### FLT3 ligand differentiates Npm1^cA^/Ptpn11^E76K^ bone marrow cells into monocytes and DCs that have an altered cytokine response to CpG.

**Figure 7:**
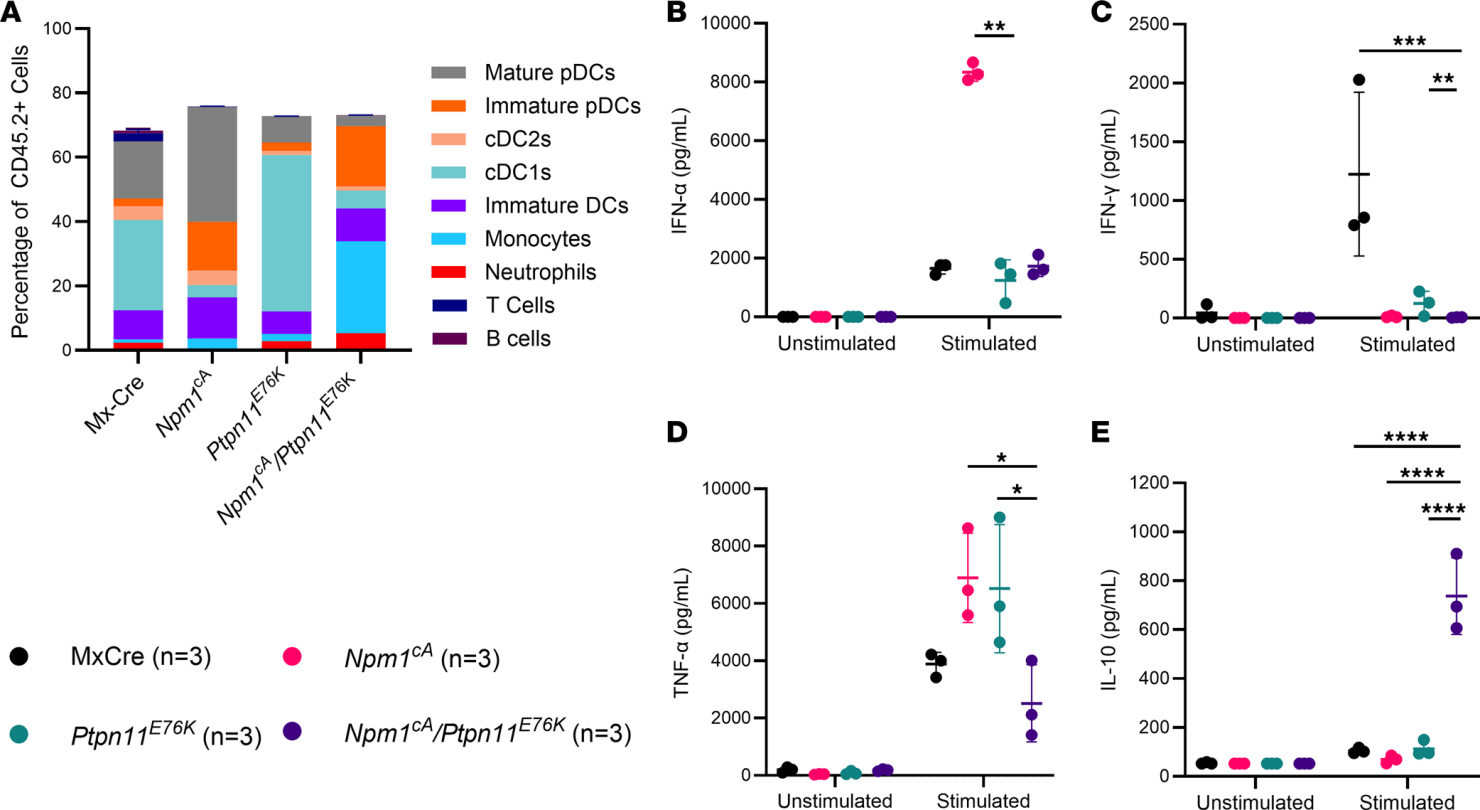
Murine *Npm1*^cA^/*Ptpn11*^E76K^ bone marrow DC differentiation and in vitro CpG stimulation. (**A**) Immune subsets of bone marrow cells from Mx-Cre (*n* = 3), *Npm1*^cA^ (*n* = 3), *Ptpn11*^E76K^ (*n* = 3), and *Npm1*^cA^/*Ptpn11*^E76K^ (*n* = 3) mice after 9 days of differentiation with FLT3 ligand in vitro. (**B**) IFN-α (pg/mL), (**C**) IFN-γ (pg/mL), (**D**) TNF-α (pg/mL), and (**E**) IL-10 (pg/mL) in the supernatant of DC-differentiated Mx-Cre (*n* = 3), *Npm1*^cA^ (*n* = 3), *Ptpn11*^E76K^ (*n* = 3), and *Npm1*^cA^/*Ptpn11*^E76K^ (*n* = 3) bone marrow cells after overnight stimulation with 0.1 μg/μL of CpG. Data are presented as mean ± SD. Statistical analysis by 1-way ANOVA with Benjamini-Hochberg FDR correction applied. *FDR-adjusted *P* ≤ 0.05, **FDR-adjusted *P* ≤ 0.01, ***FDR-adjusted *P* ≤ 0.001, ****FDR-adjusted *P* ≤ 0.0001.

Given the expansion of a DC population in these mice, we wanted to determine whether the leukemic cells isolated from *Npm1*^cA^/*Ptpn11*^E76K^ mice can respond to antigen by stimulating *Npm1*^cA^/*Ptpn11*^E76K^ bone marrow cells with CpG. We first differentiated the cells for 9 days in vitro with FLT3 ligand to ensure that there was a sufficient population of DCs, particularly in the Mx-Cre mice where these cells are more limited. The Mx-Cre control mice developed mainly mature pDCs and cDC1s, with a smaller proportion of cDC2s, immature pDCs, immature DCs, and very few monocytes. The *Npm1*^cA^ bone marrow cells developed into mainly mature pDCs, while the *Ptpn11*^E76K^ bone marrow cells differentiated into mainly cDC1s. Finally, the *Npm1*^cA^/*Ptpn11*^E76K^ bone marrow cells differentiated into monocytes, followed by immature pDCs and immature DCs (Figure 7A). After overnight stimulation with CpG, the *Npm1*^cA^/*Ptpn11*^E76K^ cells produced an equal amount of IFN-α compared to Mx-Cre controls but less IFN-γ and TNF-α (Figure 7, B–D). Compared with Mx-Cre, *Npm1*^cA^, and *Ptpn11*^E76K^ controls, the *Npm1*^cA^/*Ptpn11*^E76K^ cells produced more immunosuppressive IL-10 after CpG stimulation (Figure 7E). These results suggest that the bone marrow cells from *Npm1*^cA^/*Ptpn11*^E76K^ mice can be differentiated into monocytes and immature DCs with a potentially immunosuppressive phenotype.

## Discussion

Using scDNA-seq of AML patient samples, we present data that demonstrate for the first time to our knowledge that *PTPN11* can act as an early driver mutation in AML when accompanied by a strong oncogene such as *NPM1* mutation or rearrangement of *EVI1* or *KMT2A*. The co-occurring single-cell surface immunophenotype showed that most myeloid cells, independent of immature or mature myeloid marker expression, had leukemic mutations. These data highlight that there are few WT myeloid cells irrespective of differentiation state at diagnosis in *PTPN11*-mutated AML. We further characterized the immunophenotype of leukemic myeloid cells with spectral flow cytometry and identified variably differentiated monocytes and DCs. We then developed a transgenic mouse model concurrently expressing the *Ptpn11*^E76K^ and *Npm1*^cA^ alleles to perform validation studies and further characterize the role of a strong driver gene (mutant *NPM1*) together with a *PTPN11* mutation in driving propagation of disease. Mice harboring both *Ptpn11*^E76K^ and *Npm1*^cA^ mutations rapidly developed a fully penetrant lethal myeloid neoplasm that recapitulates many of the features of *PTPN11*-mutated AML in humans. Primary transgenic *Npm1^cA^/Ptpn11^E76K^* mice present with an expansion of variably differentiated myeloid cells, including monocytes and DCs. This diverse immunophenotype was stable upon engraftment, and all immune populations could be derived from the LSK cells. When differentiated in culture with FLT3 ligand, the leukemic cells from the *Npm1^cA^/Ptpn11^E76K^* mice could be pushed toward a DC lineage that exhibits an altered cytokine response to CpG oligodeoxynucleotide stimulation, with increased IL-10 release suggestive of an immunosuppressive phenotype.

Prior work from our group showing a high VAF of *PTPN11* mutations in some patients with AML suggested that *PTPN11* mutations may be an early co-event in leukemogenesis. The findings of our scDNA-seq analysis corroborate this, confirming that *PTPN11* can be both a late acquired subclonal mutation or an early acquired driving mutation together with another strong oncogene (*EVI1*, *KMT2A*, or *NPM1* mutation). However, a few limitations of the scDNA-seq technology utilized hindered our ability to fully understand the clonal evolution in these patients. First, we are unable to integrate information about chromosomal rearrangements with genomic mutations at a single-cell level. This was particularly pertinent in the *PTPN11*-mutated patients without a concurrent *NPM1* mutation, who frequently carried chromosome rearrangements but had few co-occurring mutations based on the bulk sequencing analysis. In these patients, we could not infer the order of acquisition of the *PTPN11* mutation relative to the chromosome rearrangement. Secondly, due to the limited coverage of the scDNA-seq panel, multiple high-VAF mutations identified in the bulk sequencing were not captured in the single-cell data. Therefore, we can only infer the order of acquisition for the captured mutations. Conversely, the scDNA-seq reliably captured some low-frequency mutations not identified in the bulk sequencing, including *NRAS*, *KRAS*, and *WT1* mutations. Lastly, while single-cell sequencing allows us to look at an individual cell, there may be small clones missed due to cellular capture and allelic dropout limitations of the MissionBio Tapestri platform. We manually analyzed each sample to mitigate these limitations. Despite these limitations, our data support the notion that a *PTPN11* mutation can occur early in AML development.

The *Npm1*^cA^*/Ptpn11*^E76K^ mouse model described herein consistently develops an AML phenotype, as evidenced by accumulation of myeloid cells in the peripheral blood and progressive anemia, thrombocytopenia, and leukocytosis at time of death. The *Npm1*^cA^/*Ptpn11*^E76K^ model has a median survival of approximately 4 months from induction, which is shorter than the *Npm1*^cA^*/Dnmt3A*^R878H^ model (30), longer than the *Npm1*^cA^/*Flt3*^ITD^ model, and comparable to the *Npm1*^cA^/*Nras*^G12D^ model (31). In addition to similar disease kinetics, the *Npm1*^cA^/*Ptpn11*^E76K^ and *Npm1*^cA^/*Nras*^G12D^ models also share a propensity for an AML with maturation immunophenotype. One limitation of the *Npm1*^cA^/*Ptpn11*^E76K^ mouse model is that it is only able to inform about the biology of early *PTPN11* mutations and not subclonal/late *PTPN11* mutations. Comprehensive immune profiling of splenocytes showed considerable diversity in the immunophenotype of *Npm1*^cA^*/Ptpn11*^E76K^ mice. *PTPN11* mutations have been previously associated with a monocytic phenotype (21), and we indeed identified a significant expansion of a population of mature and immature monocytic cells. However, we also identified expansion of cells with a mature immunophenotype, including an expansion of cDC2s and mature pDCs as well as partially differentiated DCs and pDCs. Furthermore, we found that when engrafting bulk splenocytes, mature myeloid populations were consistently present in both immunodeficient primary and secondary recipients and importantly could be derived from the LSK stem/progenitor population. Interestingly, engraftment of LSK cells was more inconsistent than unselected splenocytes from the same donors, suggesting contribution of the more mature myeloid cell populations in efficient pathogenesis of *Ptpn11*^E76K^/*Npm1*^cA^ AML. Finally, we found that isolated CD11c^+^ cells were capable of engraftment and the establishment of a lethal leukemia with equivalent efficiency to a total CD45^+^ sorted population. This supports the hypothesis that in this mouse model, leukemia-initiating cells are not exclusively found within the LSK population. While the canonical immunophenotypic definition of hematopoietic stem cells excludes CD11c-expressing cells, it has been suggested that stem cells may express CD11c (32). Our immunophenotyping data also show that multiple AML populations express CD11c, including those that express the additional markers that define DCs but also a population of cells lacking MHCII that we identified as immature DCs. Further work is needed to delineate the specific CD11c^+^ population responsible for engraftment and how the mature immune populations that comprise the AML contribute to the engraftment of LSK cells.

When comparing the populations of myeloid cells across patient samples and the murine model, we were able to identify similar populations of mature and immature monocytes, cDCs, and immature DCs that expressed CD11c but not terminally differentiated DC markers. While monocytes were not significantly upregulated in our patient samples, the scDNA-seq data indicate that these are leukemic monocytes. One major difference is that we were not able to identify a population of neutrophils in the patient samples, which is likely related to a difference in cell processing as the human blood and bone marrow samples were mononuclear cells isolated by density gradient centrifugation, whereas the mouse cells were not. Finally, we looked for the presence of pDCs in the human samples, which were present in a couple of samples but were not above the 2%-of-cells threshold to be defined as pDC AML (33). The pDC expansion in the murine model is likely related to a combination of Mx-Cre driving the expression of mutated *Ptpn11* and *Npm1* in all hematopoietic stem cells and to the fact that primary AML samples tend to have mutations in more genes than *PTPN11* and *NPM1*. These additional mutations could interfere with an expansion of pDCs.

While CD11c has been recognized as being aberrantly expressed on AML cells for many years (34, 35), DC AML is a very rare entity (36). However, it has been noted that patients with internal tandem duplication of *FLT3* (*FLT3*-ITD) have elevated numbers of cDCs and pDCs. Interestingly, Rickmann et al. noted that the intensity of expression of HLA-DR was decreased in *FLT3*-ITD–positive samples compared with *FLT3*-ITD–negative samples (37). This same group published a follow-up paper demonstrating a higher frequency of precursor DCs, defined as lineage^–^HLA-DR^+^CD11c^+^CD123^+^, and a decreased frequency of terminally differentiated DCs, defined as CD1c^+^, CD141^+^, or CD303^+^ DCs (38). These results are similar to ours, as we detected an expansion of immature DCs that are CD11c^+^HLA-DR^+^ but lack CD1c and CD141 expression. However, none of the patients in our cohort had an alteration in *FLT3*. To our knowledge, we are the first to describe an expansion of variably matured DCs in *PTPN11*-mutated AML and can confirm using scDNA-seq that these cells have leukemic mutations. While CpG stimulation in vitro resulted in altered IFN production in both murine and primary *PTPN11*- and *NPM1*-mutated AML, more work is needed to understand the full functionality of these cells and how they are contributing to leukemogenesis and treatment resistance.

In conclusion, we have demonstrated with both human and murine genetic studies that mutated *PTPN11* can have a divergent clonal hierarchy and can be a co-initiating factor with other strong oncogenes. Our murine model of *Npm1*- and *Ptpn11*-mutated AML can serve to study the role of DC expansion in both disease progression and immunosuppression associated with this disease. Previous work from Loberg et al. (30) has shown how order of acquisition of *DNMT3A* and *NPM1* mutations can impact disease trajectory. Our work provides the foundation for future studies regarding how mutated NPM1 and SHP2 proteins interact to drive the disease.

## Methods

### Sex as a biological variable.

Our study examined male and female primary samples and animals, and sex-dimorphic effects are reported.

### Patient samples.

scDNA-seq was performed on samples from 13 newly diagnosed patients from the Alliance for Clinical Trials in Oncology with de novo AML and *PTPN11* mutations. Five samples were peripheral blood and 8 were bone marrow samples. All patients had a *PTPN11* mutation as a dominant mutation (VAF > 0.3), with 9 patients having a co-occurring mutation in the *NPM1* gene. Flow cytometry for immunophenotyping was performed on 15 primary AML patient samples, of which 5 had *PTPN11*-only mutations (5 peripheral blood and 2 bone marrow samples) and 10 had *PTPN11* and *NPM1* mutations (10 peripheral blood and 6 bone marrow samples). Five healthy donor peripheral blood and 3 bone marrow samples were used as controls for the flow cytometry.

### Sequencing reagents.

The TotalSeq-D Heme Oncology Panel of oligo-conjugated reagents was purchased from BioLegend (clone information can be found in Supplemental Methods). Primers for the Myeloid Clonal Evolution Panel were purchased from MissionBio (22).

### scDNA and protein library preparation and sequencing.

Cryopreserved samples were thawed and counted using the Cellaca PLX Image Cytometry System (Nexcelom). A Dead Cell Removal Kit (Miltenyi Biotec) was then used to collect live cells. Cells were recounted and resuspended in Cell Staining Buffer (MissionBio). The remaining staining, cell encapsulation, barcoding, target PCR amplification, PCR cleanup, and quantification steps were performed according to MissionBio’s Tapestri Single-Cell DNA + Protein Sequencing Protocol. Libraries were quantified using a TapeStation (Agilent) prior to pooling. Four pooled samples were run on one S1 lane on an Illumina NovaSeq at the Cincinnati Children’s Hospital Medical Center DNA Sequencing and Genotyping Core.

### Data processing.

FASTQ files were analyzed through the Tapestri Pipeline, and the subsequent output was analyzed in R as previously described (22). Briefly, reads were aligned to the hg19 genome build (39, 40) and to cell barcodes. Genotypes were called using the GATKv3.7 (41–43). Any intronic changes were removed from the analysis and only nonsynonymous variants were included. The aligned reads of all called variants were visually inspected in the Integrative Genomics Viewer (https://www.broadinstitute.org/scientific-community/software/integrative-genomics-viewer). All variants with VAFs of less than 0.01 and genotyped in less than 50% of cells were excluded from the analysis. Clones were manually examined to exclude those that have high allele dropout rates, low genotype quality, low read depth, exhibit loss of heterozygosity, or improper amplification of mutant alleles. Further filtering and mutational order were determined by current biological knowledge of clonal evolution. For each variant, the number of cells in each genotype was counted and VAF was calculated as VAF = 2n(Het) + 2n(Homo)/2[2n(Het) + 2n(Homo) + 2n(WT)], where *n* = number of cells identified with relevant mutation. Cells with missing genotyping calls were excluded. A comparison of the bulk and single-cell sequencing VAF is presented in Supplemental Table 1. For the protein analysis, normalization was performed using DSB normalization (44). Dimension reduction, differential expression markers, and data visualization was performed using Seurat in R, as previously described (22, 45). To generate unsupervised heatmaps of protein expression in R, the ComplexHeatmap package from Bioconductor was used (46, 47).

### Primary AML sample flow cytometry.

For the primary AML immunophenotyping flow cytometry, cryopreserved peripheral blood and bone marrow cells were thawed and 1 × 10^6^ cells were obtained for staining. Cells were first stained with a Live/Dead Fixable Blue Dead Cell Stain Kit (Thermo Fisher Scientific) for 30 minutes. Cells were then washed and blocked with BD Pharmingen Human BD Fc Block (BD Biosciences) and True-Stain Monocyte Blocker (BioLegend) for 5 minutes. Antibody master mix (Supplemental Methods) with Brilliant Stain Buffer Plus (BD Biosciences) in FACS buffer (PBS, 2% FBS, and 0.1% sodium azide) was then added and cells were incubated for 30 minutes. Cells were then washed 3 times and flow cytometry data were immediately acquired on a Cytek Aurora (Cytek Biosciences) with 100,000 events collected per sample.

### Experimental animals.

*Ptpn11*^E76K^ and *Npm1*^cA^ mice were gifts from Cheng-Kui Qu (18) at Emory University (Atlanta, Georgia, USA) and George Vassiliou (27) from Cambridge Stem Cell Institute (Cambridge, United Kingdom), respectively. B6.Cg-Tg(Mx1-cre)1Cgn/J mice (referred to as Mx-Cre mice; strain 003556) (28) were purchased from The Jackson Laboratory. NOD-*Prkdc^em26Cd52^Il2rg^em26Cd22^*/NjuCrl (referred to as NCG mice; strain 572) were purchased from Charles River Laboratories. To induce expression of the transgenes, mice were injected 5 times (once every other day) with 20 μg/g of poly(I:C) (Sigma Aldrich) via intraperitoneal injection at 6–10 weeks old. Mice were bled biweekly to monitor disease development via complete blood counts (CBCs) obtained on the Element HT5 (Heska) and signs of early removal criteria (ERC). ERC was defined as 20% weight loss, partial or full hind limb paralysis, dehydration, anorexia, anemia, hunched posture, inactivity, lethargy, difficulty breathing, or rough hair coat.

### Murine cell isolation.

When a mouse met ERC, spleen, femurs, and tibias were collected in PBS and immediately processed. Spleens were dissociated in a gentleMACS Dissociator (Miltenyi Biotec) and bone marrow cells were isolated via centrifugation. Both splenocytes and bone marrow cells underwent red blood cell (RBC) lysis with 1× RBC Lysis buffer (Thermo Fisher Scientific). Samples were then sterilely cryopreserved.

### Murine peripheral blood and splenocyte flow cytometry.

For the peripheral blood flow cytometry, 20 μL of blood was blocked with TrueStain FcX (anti–mouse CD16/32) (BioLegend) and True-Stain Monocyte Blocker (BioLegend) for 5 minutes prior to staining with the 20-antibody cocktail (Supplemental Methods) in Brilliant Stain Buffer Plus (BD Biosciences) and FACS buffer. After 30 minutes of incubation, samples were lysed twice with 1× RBC Lysis Buffer. Fifty thousand events per sample were immediately collected on the Cytek Aurora.

For the splenocyte immunophenotyping flow cytometry, cryopreserved splenocytes were thawed and 1 × 10^6^ were obtained for staining. Cells were first stained with the Live/Dead Fixable Blue Dead Cell Stain Kit for 30 minutes. Cells were then washed and blocked with TrueStain FcX (anti-mouse CD16/32) and True-Stain Monocyte Blocker for 5 minutes. Antibody master mix for the splenocyte panel (Supplemental Methods) with Brilliant Stain Buffer Plus in FACS buffer was then added and cells were incubated for 30 minutes. Cells were then washed 3 times prior to paraformaldehyde fixation for 20 minutes. Cells were then washed and stored at 4°C overnight. Flow cytometry data were acquired the next day on the Cytek Aurora, with 100,000 events per sample being collected.

### In vivo bulk splenocyte, LSK, and lineage^+^ engraftment.

One million bulk splenocytes from *Ptpn11*^E76K^/*Npm1*^cA^ mouse spleens were injected via the tail vein into NCG mice. Splenocytes from these primary recipients were collected as described above and 1 × 10^6^ cells were subsequently transplanted into a secondary NCG recipient. For the LSK and lineage^+^ engraftment, splenocytes were sorted on the BigFoot Spectral Cell Sorter (Thermo Fisher Scientific), and LSKs or lineage^+^ populations (total CD45^+^, CD11c^+^ [CD45^+^CD3^–^CD19^–^Nk1.1^–^Ly6G^–^CD11c^+^] or CD11c^–^CD11b^+^ [CD45^+^CD3^–^CD19^–^Nk1.1^–^Ly6G^–^CD11c^–^CD11b^+^]) were collected for engraftment (Supplemental Methods and Supplemental Figures 7 and 8). Five thousand to 30,000 LSKs were engrafted per NCG mouse via tail vein injection. For each lineage^+^ donor, between 1 and 6 NCG recipients were engrafted per population with 3 × 10^5^ sorted cells. Disease development was monitored through weekly bleeds via CBCs and flow cytometry for CD45.2 (clone 104; BD Biosciences, 561875) and CD45.1 (clone A20; BD Biosciences, 553775). At ERC, splenocytes and bone marrow cells were cryopreserved as described above.

### In vitro murine bone marrow DC differentiation.

Cryopreserved bone marrow samples from age-matched Mx-Cre, *Npm1*^cA^, *Ptpn11*^E76K^, and *Npm1*^cA^/*Ptpn11*^E76k^ mice were thawed and cultured for 9 days in RPMI 1640 supplemented with 10% fetal calf serum (Gibco), 1 mM sodium pyruvate (Gibco), 1× MEM non-essential amino acids (Gibco), 10 mM HEPES (Gibco), 1× GlutaMAX (Gibco), 55 μM 2-mercaptoethanol (Gibco), 1× penicillin-streptomycin (Gibco), and 100 ng/mL FLT3 ligand (Peprotech). Cells were then collected either for flow cytometry or CpG stimulation. Cells collected for flow cytometry were stained with the Live/Dead Fixable Blue Dead Cell Stain Kit for 30 minutes. Cells were then washed and blocked with TrueStain FcX (anti–mouse CD16/32) and True-Stain Monocyte Blocker for 5 minutes. Antibody master mix (Supplemental Methods) with Brilliant Stain Buffer Plus in FACS buffer was then added and cells were incubated for 30 minutes. Cells were washed 3 times with FACS buffer and flow cytometry data were acquired immediately on the Cytek Aurora.

### In vitro murine bone marrow CpG stimulation.

After 9 days of growth in vitro as described above, bone marrow cells from age-matched Mx-Cre, *Npm1*^cA^, *Ptpn11*^E76K^, and *Npm1*^cA^/*Ptpn11*^E76k^ mice were resuspended at 1 × 10^6^ cells/mL and cultured overnight with 0.1 μg/μL of CpG (ODN 1585, Invivogen). Supernatant was collected and stored at –80°C. Supernatant was then thawed and cytokines were analyzed using the Mouse Anti-Virus Response LEGENDplex Panel (BioLegend). Samples were run on the Cytek Auroa, and 4,000 events were collected per sample.

### Statistics.

Data are presented as mean ± SD, unless otherwise specified. One-way ANOVA for log(%) was used to compare the frequency of various cell populations from the broad immunophenotyping panels for both primary AML samples and murine splenocytes. One-way ANOVA for log(weight) or log(count) using contrast for each pair of groups was used to determine differences in spleen weight, WBC, RBC, and platelet (PLT) counts at ERC. One-way ANOVA for log(concentration) using contrast for each pair of groups was used for cytokine production analysis. The Shapiro-Wilk method was used for normality. For multiple-comparisons, the Benjamini-Hochberg method was used to adjust *P* values. To analyze differences in overall survival time, we fit a Cox’s proportional hazards model for group and used contrast to compare each pair of groups. A *P* value of less than 0.05 was used to determine significance.

### Study approval.

All patients provided written informed consent prior to participation in the following protocols for the collection of pretreatment AML peripheral blood and bone marrow samples: CALGB 8461 (cytogenetic studies only, no tissue obtained), CALGB 9665 (leukemia tissue bank, blood and bone marrow obtained for this), and CALGB 20202 (patient consent to perform molecular studies). All protocols were approved by the Institutional Review Board at each center. For the healthy donor bone marrow samples, all donors provided written informed consent prior to participation, and protocols were approved by the IRB at the University of Cincinnati (UC). Healthy donor peripheral blood samples were purchased from the Hoxworth Blood Center (UC). All animal experiments were conducted after approval by UC and The Ohio State University (OSU) Institutional Animal Care and Use Committees (IACUC). Both male and female mice were used. No work was done related to this paper at the University of Pittsburgh (JCB moved here after acceptance of this paper).

### Data availability.

All supporting data including means and SD reported in the text are available in the Supporting Data Values file which can be found in the Supplemental Material. Raw sequencing data are available in NCBI’s SRA (SUB15641400). Individuals desiring data related to this work can correspond with one of the corresponding authors.

## Author contributions

SF and CS have equally contributed to the design and execution of the presented data and preparation and revision of the manuscript and therefore were designated as co-first authors. SF, CS, EH, and JCB conceived and designed the study. SF, CS, KB, CC, CF, ML, KF, BW, JRL, and TMS performed experiments. SF, CS, KQ, LAM, and RLB analyzed the single cell sequencing data. SF and CS analyzed all non-sequencing data. CP provided experimental methods for dendritic cell differentiation. NZ helped with immunophenotype definitions. KM, DN, and AKE provided patient samples and corresponding demographics, cytogenetics, and outcomes. JP and SNR performed the statistics. WMY and CKQ provided the *Ptpn11*^E76K^ mouse. SF, CS, EH, and JCB wrote the original draft of manuscript. MEJ and JPP contributed to experimental design. AC, RS, ESW, JK, and BP were involved in the Alliance studies that collected the primary samples used in this manuscript. All authors reviewed and edited the manuscript. JCB and EH obtained funding for this study. EH and JCB supervised this study.

## Funding support

This work is the result of NIH funding, in whole or in part, and is subject to the NIH Public Access Policy. Through acceptance of this federal funding, the NIH has been given a right to make the work publicly available in PubMed Central.

- NIH grants F30CA265281 (to SF), R35CA197734 and UG1 CA233338 (to JCB).
- Department of Defense grants HT9425-24-1-0821 (to EH).
- Pelotonia graduate fellowship (to SF).
- National Cancer Institute grant R00CA252005 (to LAM).
- American Society of Hematology Junior Faculty Scholar award (to LAM).
- NIH grant P30CA016058 (to The Ohio State University Comprehensive Cancer Center Leukemia Tissue Bank Shared Resource).

## Supplementary Material

Supplemental data

Supporting data values

## Figures and Tables

**Table 1:**
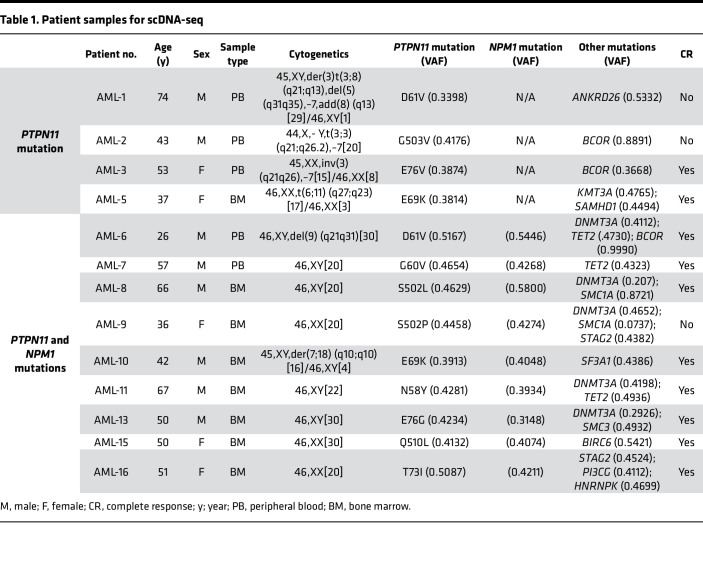
Patient samples for scDNA-seq

## Version 1 02/23/2026

Electronic publication
