# Supplementary material for: Characterization of the clonal hierarchy and immunophenotype of PTPN11 mutations in acute myeloid leukemia
Source: JCI Insight. 2026 Feb 23;11(4):e193779. doi: 10.1172/jci.insight.193779 (PMC12956013; doi:10.1172/jci.insight.193779)
Supplement: Supplemental data [file jciinsight-11-193779-s070.pdf]

Supplemental Methods

TotalSeq Heme Oncology Cocktail Clones

CD64 (clone 10.1), CD34 (clone 581), CD90 (clone 5e10), CD117 (clone 104D2), CD304 (clone 12C2), CD303 (clone 201A), CD45 (clone 2D1), CD16 (clone 3G8), CD56 (clone 5.1H11), CD123 (clone 6H6), CD49d (clone 9F10), FcεR1α (clone AER-37), CD62P (clone AK4), CD25 (clone BC96), CD30 (clone BY88), CD7 (clone CD7-6B7), CD71 (clone CY1G4). CD62L (clone DREG-56), CD69 (clone FN50), CD163 (clone GHI/61), CD83 (clone HB15e), CD45RA (clone HI100), CD10 (clone HI10a), CD19 (clone HIB19), CD38 (clone HIT2), CD11b (clone ICRF44), CD44 (clone IM7), CD1c (clone L161), HLA-DR (clone L243), CD14 (clone M5E2), CD141 (clone M80), CD138 (clone MI15), CD33 (clone P67.6), CD4 (clone RPA-T4), CD22 (clone S-HCL-1), CD11c (clone S-HCL-3), CD8 (clone SK1), CD2 (clone TS1/8), CD45RO (clone UCHL1), CD3 (clone UCHT1), CD5 (clone UCHT2), CD13 (clone WM15).

Primary AML Immunophenotyping Antibodies and Cell Surface Marker Profiles

Specificity	Clone	Manufacturer	Cat no
CD33	WM53	BD	568374
CD45	HI30	BD	569101
LIVE DEAD Blue	--	Thermo	L23105
CD99	TU12	BD	749011
CD5	UCHT2	BD	751289
CD304	MAB3	BD	756730
CD47	CC2C6	BD	753791
HLA DR	G46-6	BD	568335
CD34	QBEND/10.rMAb	BD	568861
CD4	OKT4	BioLegend	317423
CD303	V24-785	BD	748006
CD16	3G8	Biolegend	302047
CD11b	IGRF44	Biolegend	301325
CD163	GHI/61	Biolegend	333616
CD37	M-T701	BD	742400
CD83	HB15e	BioLegend	305333
CD206	19.2	BD	746891
CD90	5E10	Biolegend	328141
CD13	WM15	BD	564549
CD8	SK1	BioLegend	344760
CD14	63D3	Biolegend	367147
CD200	MRC OX-104	BD	759116
CD15	W6D3	BioLegend	323018
CD7	M-T 701	BD	566488
FceR1a	AER-37	BD	757889
CD86	2331 (FUN-1)	BD	757163
CD71	M-A712	BD	755839
CD117	S18022G	BioLegend	375205
CD1c	L161	Biolegend	331531
CD3	SK7	BioLegend	344859
CD56	B159	BD	571468
PD-1	EH12.2H7	BioLegend	329973
CD141	M80	Biolegend	344109
CD19	HIB19	Biolegend	302287
CD11c	Bu15	BioLegend	337207
CD41	HIP8	Biolegend	303725
CD38	HB7	BD	567987
CD64	10.1	BioLegend	305035
CD123	6H6	BioLegend	306053

CD34+/CD38+ cells (CD45+/CD3-/CD19-/CD7+/-/CD56+/-/CD34+/CD38+)

CD34+/CD38- cells (CD45+/CD3-/CD19-/CD7+/-/CD56+/-/CD34+/CD38-)

CD14+ Monocytes (CD45+/CD3-/CD19-/CD7+/-/CD56+/-/CD34-/CD38+/HLA-DR+/CD123-/CD14+/CD11c+/-),

pre-cDCs (CD45+/CD3-/CD19-/CD7+/-/CD56+/-/CD34-/CD38+/HLA-DR+/CD123+/CD303-/CD304-/CD11c+/CD33+)

pDCs (CD45+/CD3-/CD19-/CD7+/-/CD56+/-/CD34-/CD38+/HLA-DR+/CD123+/CD303+ and/or CD304+)

CD1c+ DCs (CD45+/CD3-/CD19-/CD7+/-/CD56+/-/CD34-/CD38+/HLA-DR+/CD123-/CD14-/CD11c+/CD1c+/CD141-)

CD41+ DCs (CD45+/CD3-/CD19-/CD7+/-/CD56+/-/CD34-/CD38+/HLA-DR+/CD123-/CD14-/CD11c+/CD1c-/CD141+)

Immature DCs (CD45+/CD3-/CD19-/CD7+/-/CD56+/-/CD34-/CD38+/HLA-DR+/CD123-/CD14-/CD11c+/CD1c-/CD141-)

Primary AML Peripheral Blood *In Vitro* CpG Stimulation

Primary AML peripheral blood samples were sterilely thawed and suspended at 1e6 cells/mL in StemSpan (StemCell) supplemented with 100ng/mL FLT3 ligand (PeproTech), 10ng/mL SCF (PeproTech), 50ng/mL TPO (PeproTech), and 1ug/mL Stemregenin (StemCell). Cells were cultured with 10ug of CpG (ODN 2216) (InvivoGen) overnight from which supernatant was collect and stored at -80°C. Supernatant was then thawed and cytokines were analyzed using the Human Anti-Virus Response Panel 1 (Biolegend). Samples were run on the Cytek Aurora (Cytek Biosciences), and 3,000 events were collected per sample.

Murine Peripheral Blood Immunophenotyping Antibodies

Specificity	Clone	Vendor	Cat no
CD8	53-6.7	BD	563786
CD45	I3/2.3	BD	752411
CD43	S7	BD	752307
CD3	145-2C11	BD	750638
I-A/I-E (MHCII)	2G9	BD	748708
CD27	LG.3A10	BD	741959
SiglecH	440c	BD	566581
Sytox Blue		Thermo	S34857
CD45R (B220)	RA3-6B2	Biolegend	103248
Ly6C	AL-21	BD	563011
CX3CR1	SA011F11	Biolegend	149033
NK1.1	PK136	BD	569723
CD44	IM7	BD	563736
CD11b	M1/70	Biolegend	101206
Ly6G	1A8	Biolegend	127654
CD19	1D3	BD	566411
CD62L	MEL-14	BD	569209
cKit	ACK2	BD	567471
CD25	PC61	BD	552880
Ly6A/E (Sca1)	D7	Biolegend	108118
CD11c	N418	Biolegend	117352
CD4	GK1.5	Biolegend	100480

Murine Splenocyte Immunophenotyping Antibodies and Cell Surface Marker Profiles

Specificity	Clone	Vendor	Cat no
CD11b	M1/70	BD	563553
Live/Dead		Thermo	L23105
CD45.2	104	BD	569670
CD23	B3B4	BD	741228
CD43	S7	BD	752307
CD24	30-F1	BD	752765
MHC II	2G9	BD	748708
CD27	LG.3A10	BD	741959
Siglec H	440c	BD	566581
CD93	AA4.1	Thermo	62589282
Ter119	TER-119	Thermo	48-5921-82
Sca1	D7	BD	746663
CD45.1	A20	BioLegend	110741
Ly6C	HK1.4	BioLegend	128030
NK1.1	PK136	BioLegend	108753
CX3CR1	SA011F11	BioLegend	149033
CD44	IM7	BD	563971
CD8a	5H10-1	BD	752630
IgM	R6-60.2	BD	564028
CD138	281-2	BD	564511
CD4	GK1.5	BioLegend	100406
CD11c	N418	BioLegend	117366
Ly6G	1A8	BioLegend	127654
CD21/35	eBio4E3 (4E3)	Thermo	46021282
cKit	ACK2	BD	567471
F4/80	QA17A29	BioLegend	157312
CD172a	P84	BioLegend	144016
CD19	6D5	BioLegend	115574
CD49b	DX5	Thermo	35-597-180
CD206	C068C2	BioLegend	141720
CD317	927	BioLegend	127016
CD3	17A2	BD	557869
IgD	11-26c.2a	BioLegend	405750
CD86	GL1	BD	565479
CD5	53-7.3	BioLegend	100634
B220	RA3-6B2	BioLegend	103277

Cell surface marker profiles were defined as:
neutrophils (CD45.2+/CD3-/CD19-/NK1.1-/Ly6G+),
macrophages (CD45.2+/CD3-/CD19-/NK1.1-/Ly6G-/CD11c-/F4-80+),
monocytes (CD45.2+/CD3-/CD19-/NK1.1-/Ly6G-/CD11c-/CD11b+/F4/80low/CX3CR1+),
cDC1s (CD45.2+/CD3-/CD19-/NK1.1-/Ly6G-/CD11c+/B220-/MHC II+/CD172a+/-/CD11b+)
cDC2s (CD45.2+/CD3-/CD19-/NK1.1-/Ly6G-/CD11c+/B220-/MHC II+/CD172a-/CD11b-/CD24+/CD8a+)
pDCs (CD45.2+/CD3-/CD19-/NK1.1-/Ly6G-/CD11c+/MHC II+/B220+).

LSK Engraftment Sorting Antibodies

Specificity	Clone	Vendor	Cat no
CD11b	M1/70	BD	563553
CD45.2	104	BD	741092
B220	RA3-6B2	BD	612838
SiglecH	440c	BD	566581
Sytox Blue		Thermo	S34857
Ly6C	HK1.4	BioLegend	128030
CD11c	N418	BioLegend	117349
CD19	1D3	BD	564509
CD3	145-2C11	BioLegend	100306
Ter119	TER-119	BioLegend	116206
FceR1	MAR-1	BioLegend	134306
NK1.1	S17016D	BioLegend	156522
Sca1	D7	BD	742089
cKit	ACK2	BD	567471
CD317	927	BioLegend	127016
Ly6G	1A8	BD	565369

Murine Bone Marrow Dendritic Cell Differentiation Flow

Specificity	Clone	Vendor	Cat no
CD11b	M1/70	BD	563553
Live/Dead		Thermo	L23105
CD24	30-F1	BD	752765
MHC II	2G9	BD	748708
Siglec H	440c	BD	566581
Sca1	D7	BD	746663
Ly6C	HK1.4	BioLegend	128030
CD8a	5H10-1	BD	752630
CD11c	N418	BioLegend	117366
Ly6G	1A8	BioLegend	127654
cKit	ACK2	BD	567471
F4/80	QA17A29	BioLegend	157312
CD172a	P84	BioLegend	144016
CD19	6D5	BioLegend	115574
CD206	C068C2	BioLegend	141720
CD317	927	BioLegend	127016
CD3	17A2	BD	557869
CD86	GL1	BD	565479
B220	RA3-6B2	BioLegend	103277

Lineage positive Engraftment Sorting Antibodies

Specificity	Clone	Vendor	Cat no
CD11b	M1/70	BD	563553
CD45.2	104	BD	741092
B220	RA3-6B2	BD	612838
CD8a	5H10-1	BD	752640
SiglecH	440c	BD	566581
Sytox Blue		Thermo	S34857
Ly6C	HK1.4	BioLegend	128030
CD11c	N418	BioLegend	117349
CD19	1D3	BD	564509
CD3	145-2C11	BioLegend	100306
Ter119	TER-119	BioLegend	116206
FceR1	MAR-1	BioLegend	134306
NK1.1	S17016D	BioLegend	156522
cKit	ACK2	BD	567471
CD317	927	BioLegend	127016
Ly6G	1A8	BD	565369
CD24	30-F1	BD	567843

Supplemental Figures

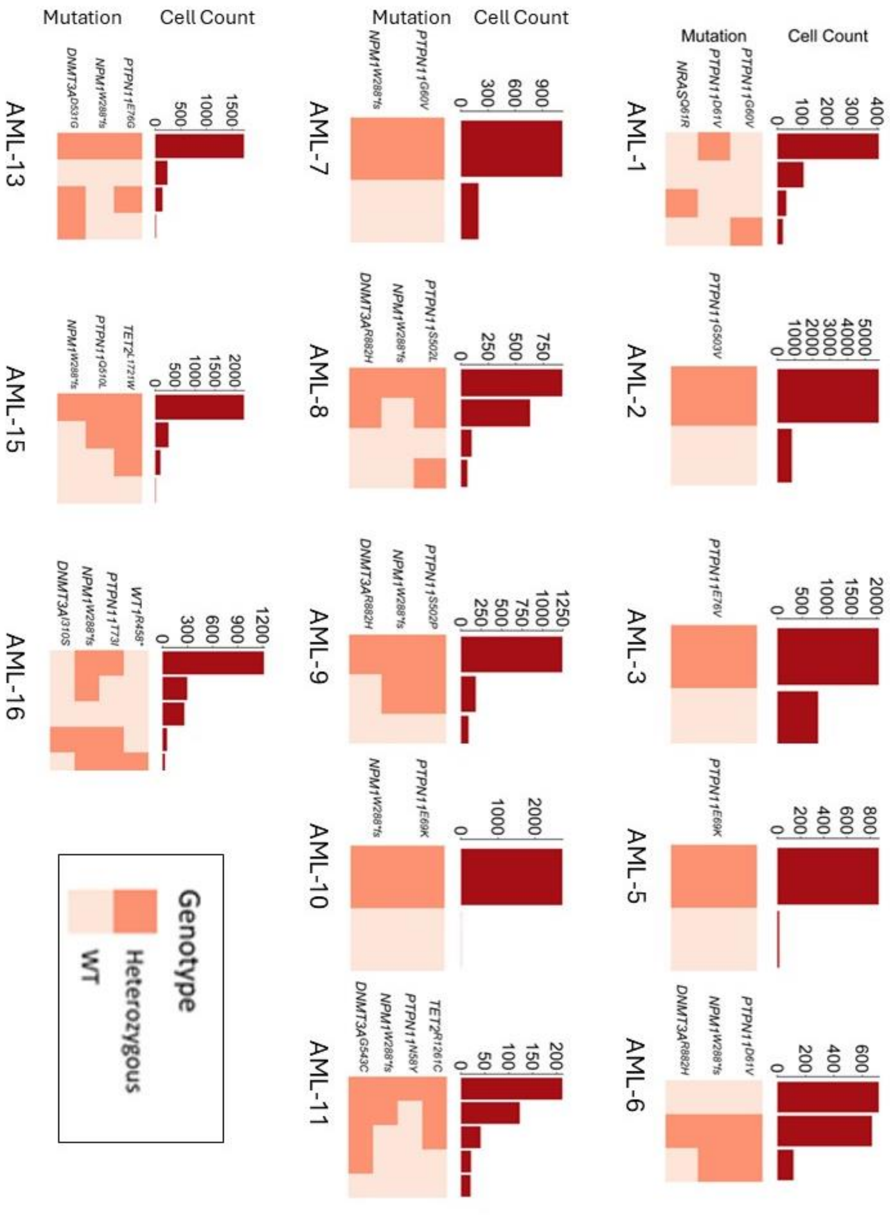

Supplemental Figure 1. Clonographs for peripheral blood and bone marrow samples from AML patients at diagnosis. For each sample, the histogram represents the number of cells within each clone ranked in decreasing frequency and the heatmap represents zygosity for each mutation with light orange being wild-type and dark orange being heterozygous.

Table 1. Comparison of variant allele frequencies identified by bulk and single-cell DNA sequencing

Patient ID	Gene	Bulk sequencing VAF	Tapestri VAF
AML1	*PTPN11*^{D61V}	0.3398	0.3726
	ANKRD26^{E1110fs}	0.5332	NC
	NRAS^{Q61R}	--	0.0496
	PTPN11^{G60V}	--	0.0209
AML-2	*PTPN11*^{G503V}	0.4176	0.4526
	BCOR^{DL442}	0.8891	NC
AML3	*PTPN11*^{E76V}	0.3874	0.4003
	BCOR$^{P142P?}$	0.3668	NC
AML5	*PTPN11*^{E69K}	0.3814	0.4675
	KMT2A^{K1211Q}	0.4765	NC
	SAMHD1^{G236V}	0.4494	NC
[redacted]			
AML6	*PTPN11*^{D61V}	0.5167	0.4311
	NPM1^{L287LC}	0.5446	0.3948
	DNMT3A^{R882H}	0.4112	0.3123
	TET2^{K1208E}	0.473	NC
	BCOR^{P838S}	0.999	NC
AML7	*PTPN11*^{G60V}	0.4654	0.4556
	NPM1^{W288CM}	0.4268	0.4439
	TET2^{C314W}	0.4323	NC
AML8	*PTPN11*^{S502L}	0.4629	0.4757
	NPM1^{W288CQ}	0.58	0.3205
	DNMT3A^{R882H}	0.207	0.4115
	SMC1A^{R398Q}	0.8721	NC
AML9	*PTPN11*^{S502P}	0.4458	0.4838
	NPM1^{L287LC}	0.4274	0.4727
	DNMT3A^{R882H}	0.4652	0.3516
	SMC1A^{R469P}	0.0737	NC
	STAG2^{L1110R}	0.4382	NC
AML10	*PTPN11*^{E69K}	0.3913	0.4769
	NPM1^{L287LC}	0.4048	0.4687
	SF3A1^{G413S}	0.4386	NC
[redacted]			
AML11	*PTPN11*^{N58Y}	0.4281	0.3522
	NPM1^{L287LC}	0.3934	0.4581
	DNMT3A^{G543C}	0.4198	0.4855
	TET2^{L230}	0.4936	0.4769

**AML13**	*PTPN11* ^{E76G}	0.4234	0.4334
	NPM1 ^{W288CM}	0.3148	0.4486
	DNMT3A ^{D342G}	0.2926	0.4466
	SMC3 ^{A244D}	0.4932	NC
**AML15**	*PTPN11* ^{Q510L}	0.4132	0.4284
	NPM1 ^{L287LC}	0.4074	0.464
	BIRC6 ^{I4153L}	0.5421	NC
	TET2 ^{L1721W}	--	0.4842
**AML16**	*PTPN11* ^{T73I}	0.5087	0.2692
	NPM1 ^{L287LC}	0.4211	0.4681
	STAG2 ^{N1223K}	0.4524	NC
	PI3CG ^{D358Y}	0.4112	NC
	HNRNPK ^{Y280}	0.4699	NC
	DNMT3A ^{I310S}	--	0.0624
	WT1 ^{R458*}	--	0.0136

NC = Variant not covered by the panel

-- = mutation not called in bulk seqencing analysis

Supp Fig 2A

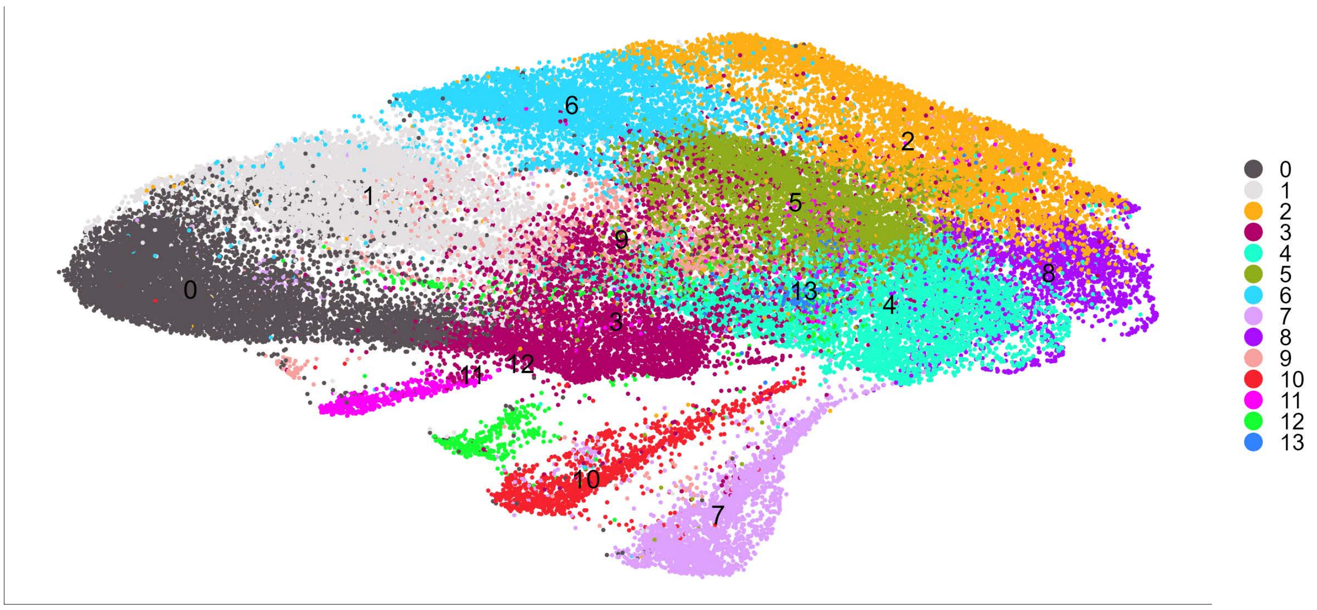

6
2
1
5
9
0
13
4
8
3
11
12
10
7
0
1
2
3
4
5
6
7
8
9
10
11
12
13

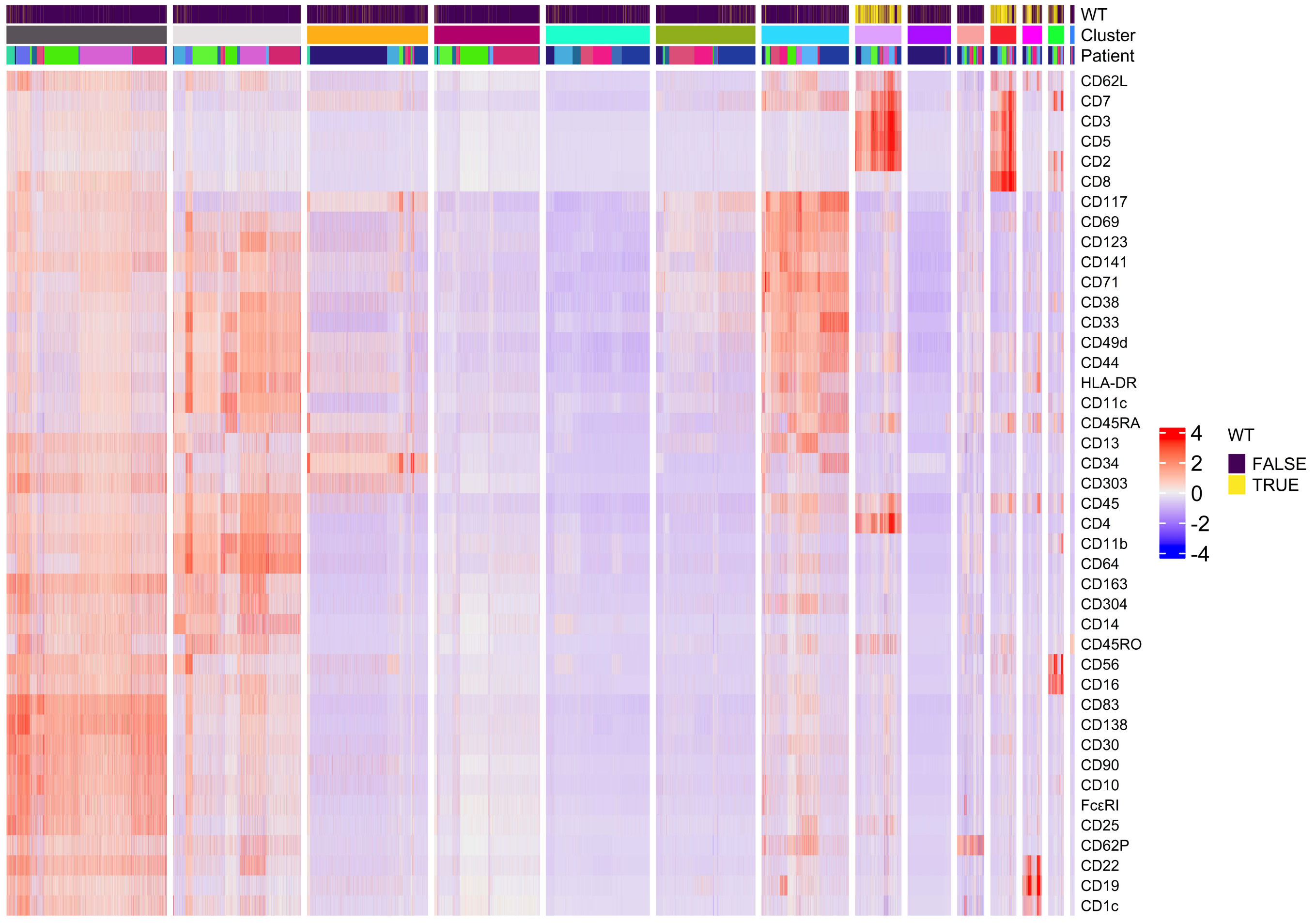

WT
Cluster
Patient
CD62L
CD7
CD3
CD5
CD2
CD8
CD117
CD69
CD123
CD141
CD71
CD38
CD33
CD49d
CD44
HLA-DR
CD11c
CD45RA
CD13
CD34
CD303
CD45
CD4
CD11b
CD64
CD163
CD304
CD14
CD45RO
CD56
CD16
CD83
CD138
CD30
CD90
CD10
FcεRI
CD25
CD62P
CD22
CD19
CD1c
4
2
0
-2
-4
WT
FALSE
TRUE

Supp Fig 2B

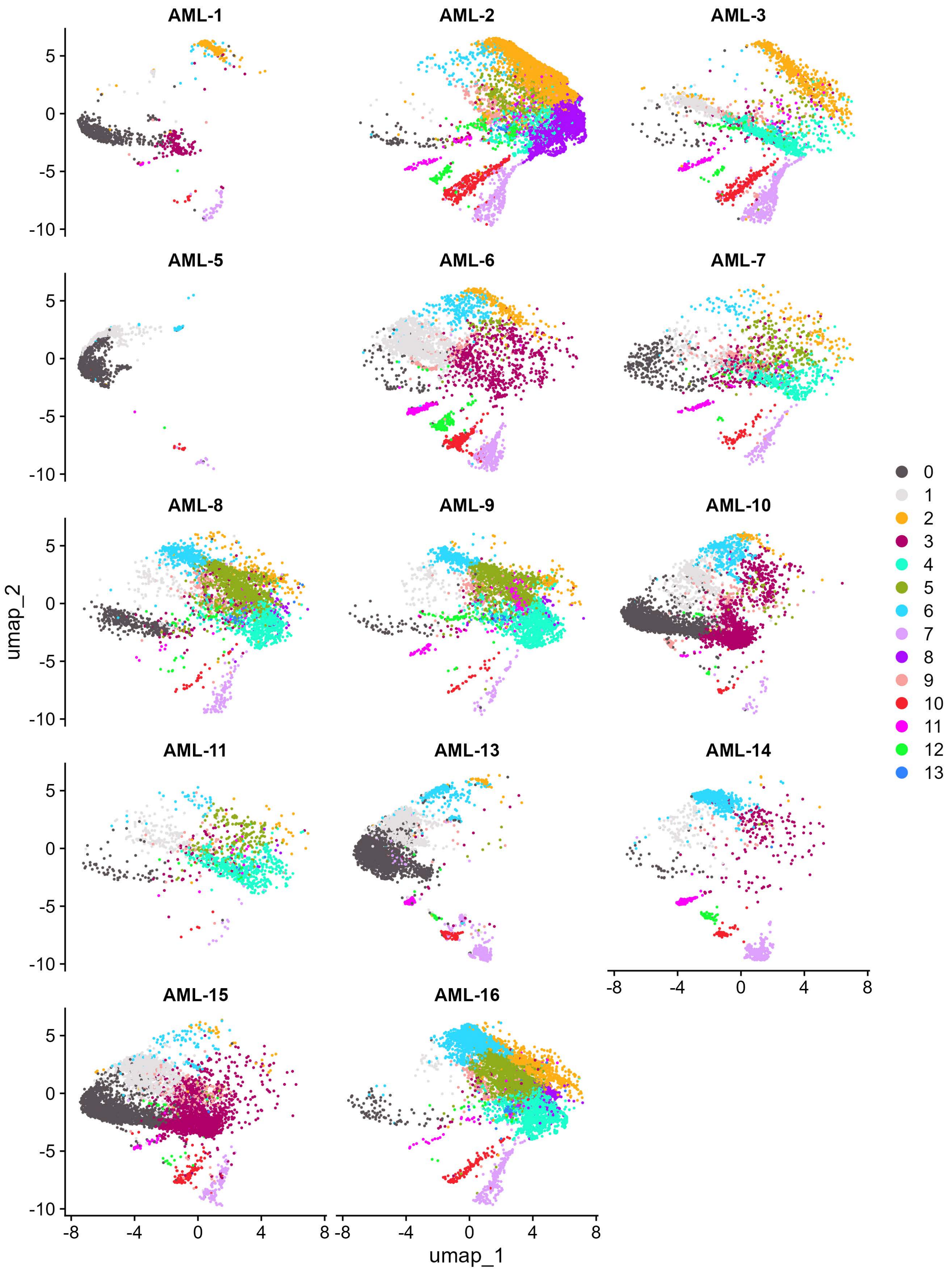
AML-1
AML-2
AML-3
AML-5
AML-6
AML-7
AML-8
AML-9
AML-10
AML-11
AML-13
AML-14
AML-15
AML-16
umap_1
umap_2
0
1
2
3
4
5
6
7
8
9
10
11
12
13

Supp Fig 2C

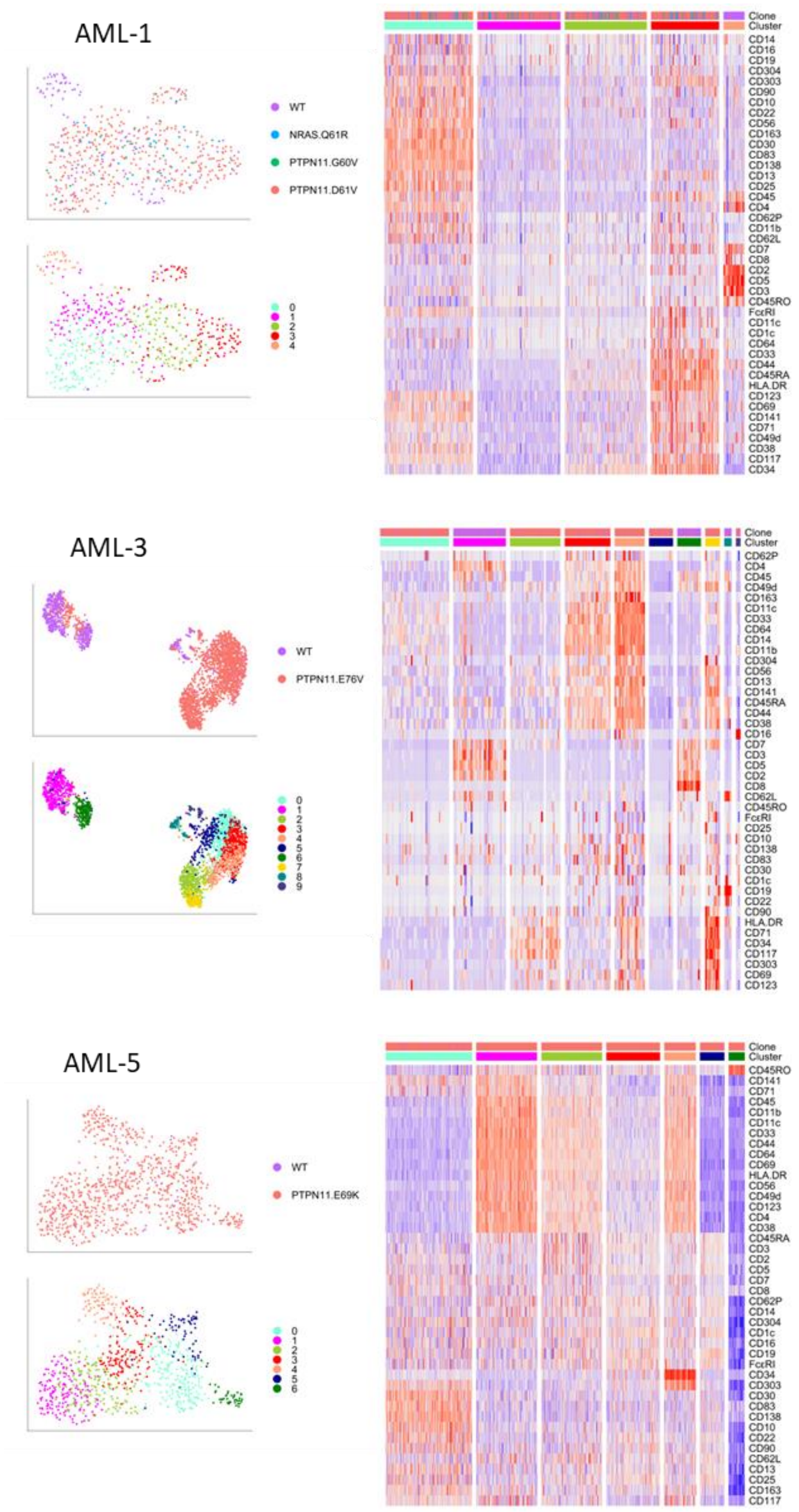
AML-1
WT
NRAS.Q61R
PTPN11.G60V
PTPN11.D61V
Clone
Cluster
AML-3
WT
PTPN11.E76V
Clone
Cluster
AML-5
WT
PTPN11.E69K
Clone
Cluster

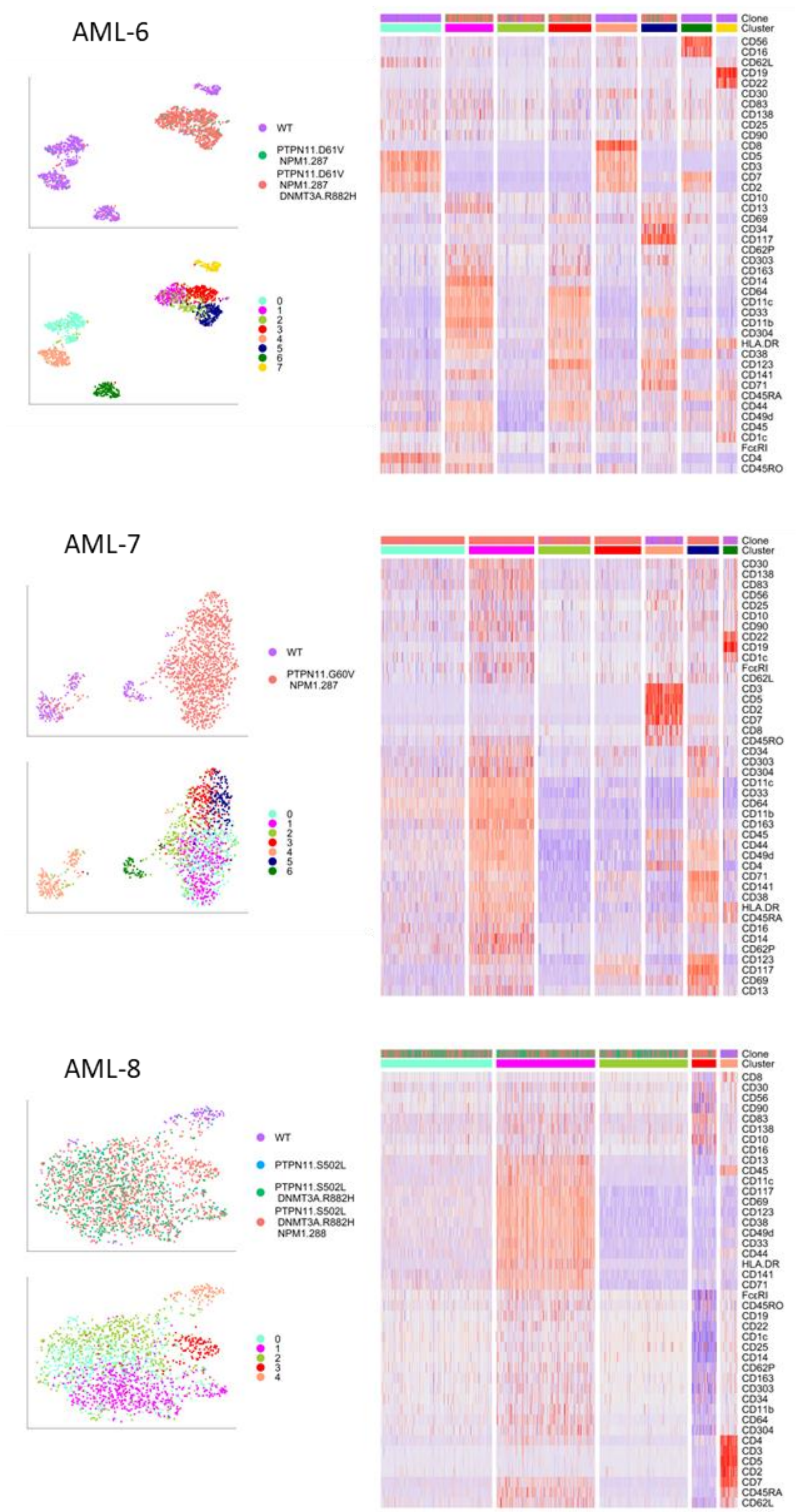

AML-6
WT
PTPN11.D61V NPM1.287
PTPN11.D61V NPM1.287 DNMT3A.R882H
0
1
2
3
4
5
6
7
Clone
Cluster
CD56
CD16
CD62L
CD19
CD22
CD30
CD83
CD138
CD25
CD90
CD8
CD5
CD3
CD7
CD2
CD10
CD13
CD69
CD34
CD117
CD62P
CD303
CD163
CD14
CD64
CD11c
CD33
CD11b
CD304
HLA.DR
CD38
CD123
CD141
CD71
CD45RA
CD44
CD49d
CD45
CD1c
FcεRI
CD4
CD45RO
AML-7
WT
PTPN11.G60V NPM1.287
0
1
2
3
4
5
6
Clone
Cluster
CD30
CD138
CD83
CD56
CD25
CD10
CD90
CD22
CD19
CD1c
FcεRI
CD62L
CD3
CD5
CD2
CD7
CD8
CD45RO
CD34
CD303
CD304
CD11c
CD33
CD64
CD11b
CD163
CD45
CD44
CD49d
CD4
CD71
CD141
CD38
HLA.DR
CD45RA
CD16
CD14
CD62P
CD123
CD117
CD69
CD13
AML-8
WT
PTPN11.S502L
PTPN11.S502L DNMT3A.R882H
PTPN11.S502L DNMT3A.R882H NPM1.288
0
1
2
3
4
Clone
Cluster
CD8
CD30
CD56
CD90
CD83
CD138
CD10
CD16
CD13
CD45
CD11c
CD117
CD69
CD123
CD38
CD49d
CD33
CD44
HLA.DR
CD141
CD71
FcεRI
CD45RO
CD19
CD22
CD1c
CD25
CD14
CD62P
CD163
CD303
CD34
CD11b
CD64
CD304
CD4
CD3
CD5
CD2
CD7
CD45RA
CD62L

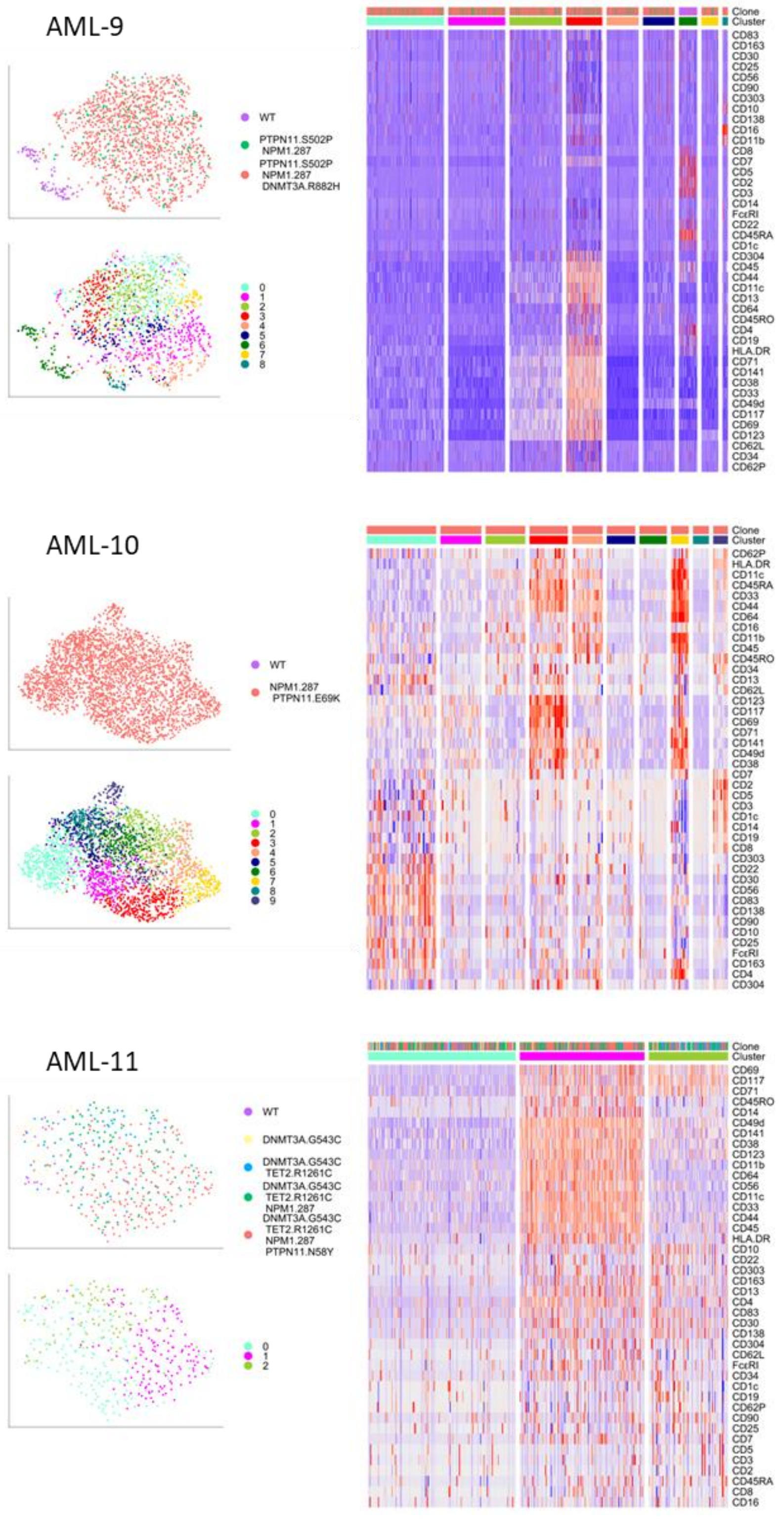
AML-9
WT
PTPN11.S502P NPM1.287
PTPN11.S502P NPM1.287 DNMT3A.R882H
0
1
2
3
4
5
6
7
8
Clone
Cluster
AML-10
WT
NPM1.287 PTPN11.E69K
0
1
2
3
4
5
6
7
8
9
Clone
Cluster
AML-11
WT
DNMT3A.G543C
DNMT3A.G543C TET2.R1261C
DNMT3A.G543C TET2.R1261C NPM1.287
DNMT3A.G543C TET2.R1261C NPM1.287 PTPN11.N58Y
0
1
2
Clone
Cluster

AML-13

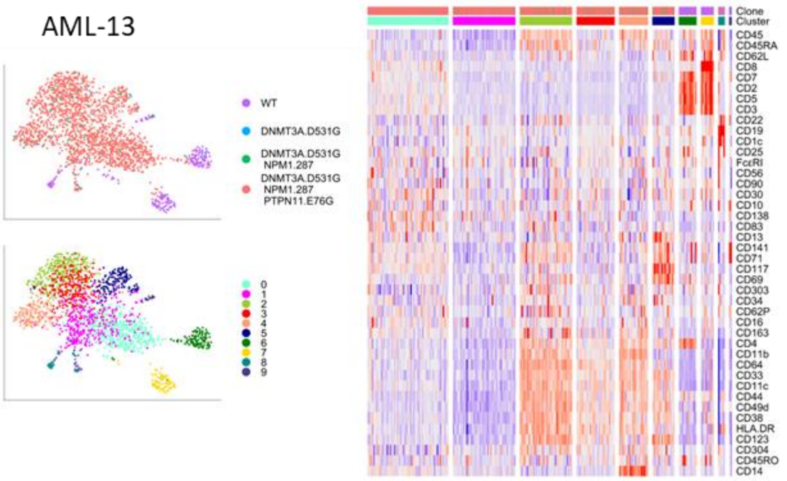
WT
DNMT3A.D531G
DNMT3A.D531G NPM1.287
DNMT3A.D531G NPM1.287 PTPN11.E76G
0
1
2
3
4
5
6
7
8
9
Clone
Cluster
CD45
CD45RA
CD62L
CD8
CD7
CD2
CD5
CD3
CD22
CD19
CD1c
CD25
FcεRI
CD56
CD90
CD30
CD10
CD138
CD83
CD13
CD141
CD71
CD117
CD69
CD303
CD34
CD62P
CD16
CD163
CD4
CD11b
CD64
CD33
CD11c
CD44
CD49d
CD38
HLA.DR
CD123
CD304
CD45RO
CD14

AML-14

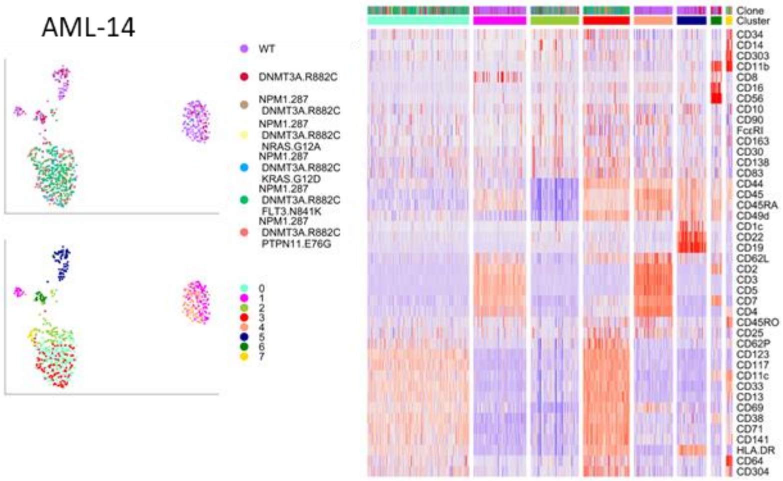
WT
DNMT3A.R882C
NPM1.287 DNMT3A.R882C
NPM1.287 DNMT3A.R882C NRAS.G12A
NPM1.287 DNMT3A.R882C KRAS.G12D
NPM1.287 DNMT3A.R882C FLT3.N841K
NPM1.287 DNMT3A.R882C PTPN11.E76G
0
1
2
3
4
5
6
7
Clone
Cluster
CD34
CD14
CD303
CD11b
CD8
CD16
CD56
CD10
CD90
FcεRI
CD163
CD30
CD138
CD83
CD44
CD45
CD45RA
CD49d
CD1c
CD22
CD19
CD62L
CD2
CD3
CD5
CD7
CD4
CD45RO
CD25
CD62P
CD123
CD117
CD11c
CD33
CD13
CD69
CD38
CD71
CD141
HLA.DR
CD64
CD304

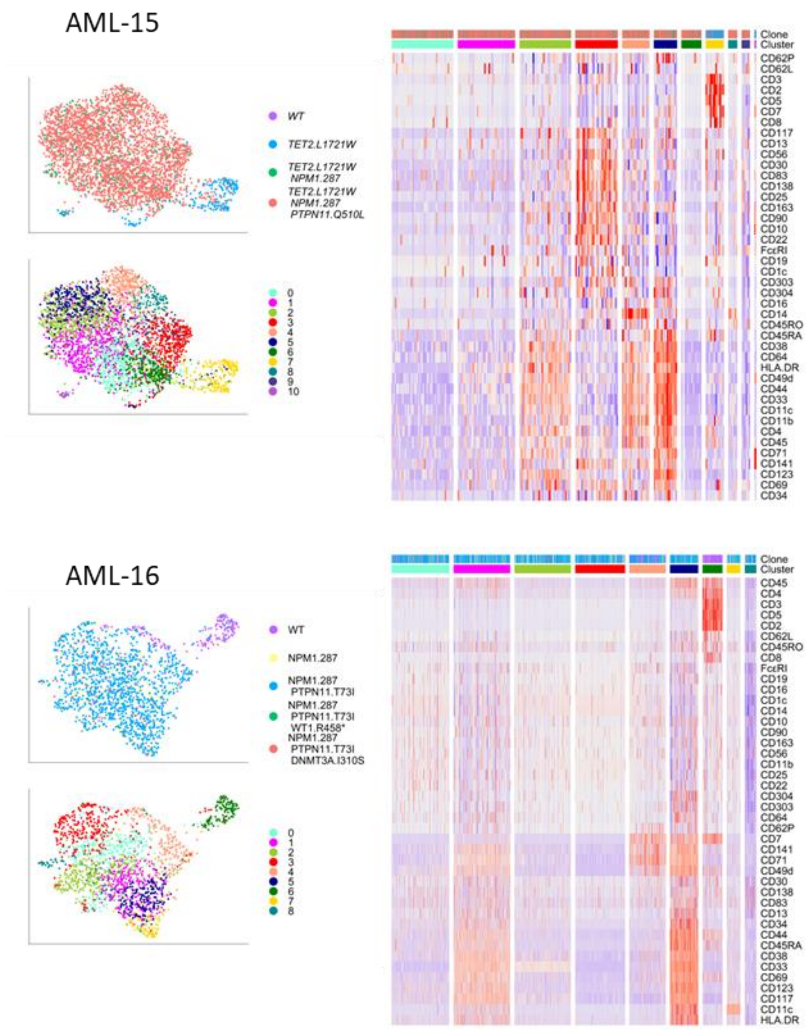

Supplemental Figure 2. Cell surface marker expression at the singe-cell based on proteomics from peripheral blood and bone marrow primary AML samples. A) For combined samples a UMAP overlayed with genotype and nearest neighbor clustering and a heatmap with select lineage defining markers. B) Individual patients projected onto combined UMAP overlayed with nearest neighbor clustering. C) Individualized UMAP overlayed with genotype and nearest neighbor clustering and a heatmap for each patient.

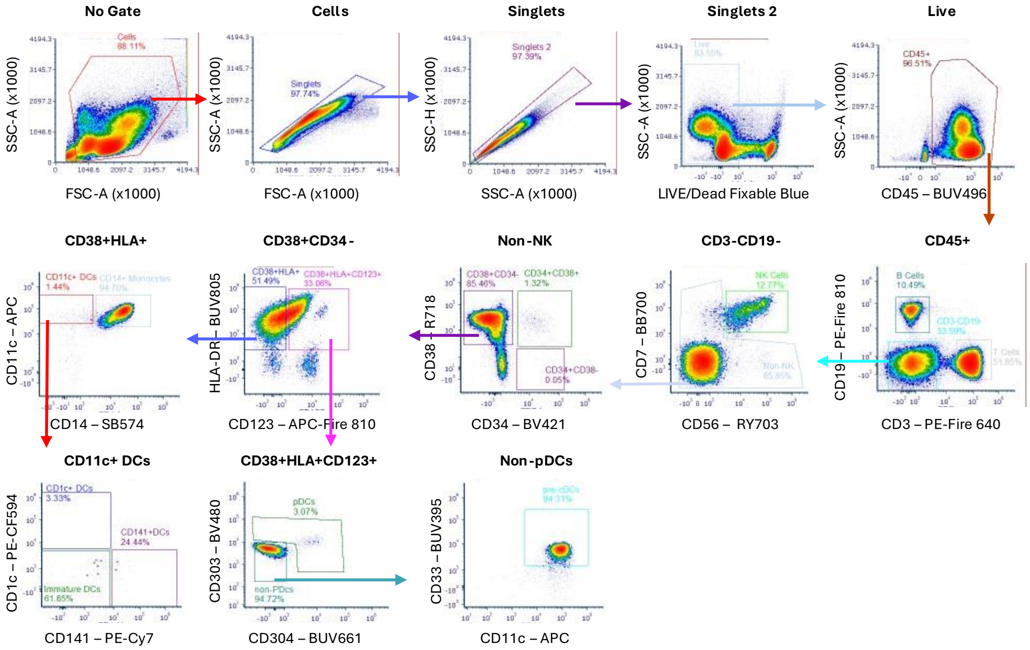

Supplemental Figure 3. Gating strategy for primary AML samples.

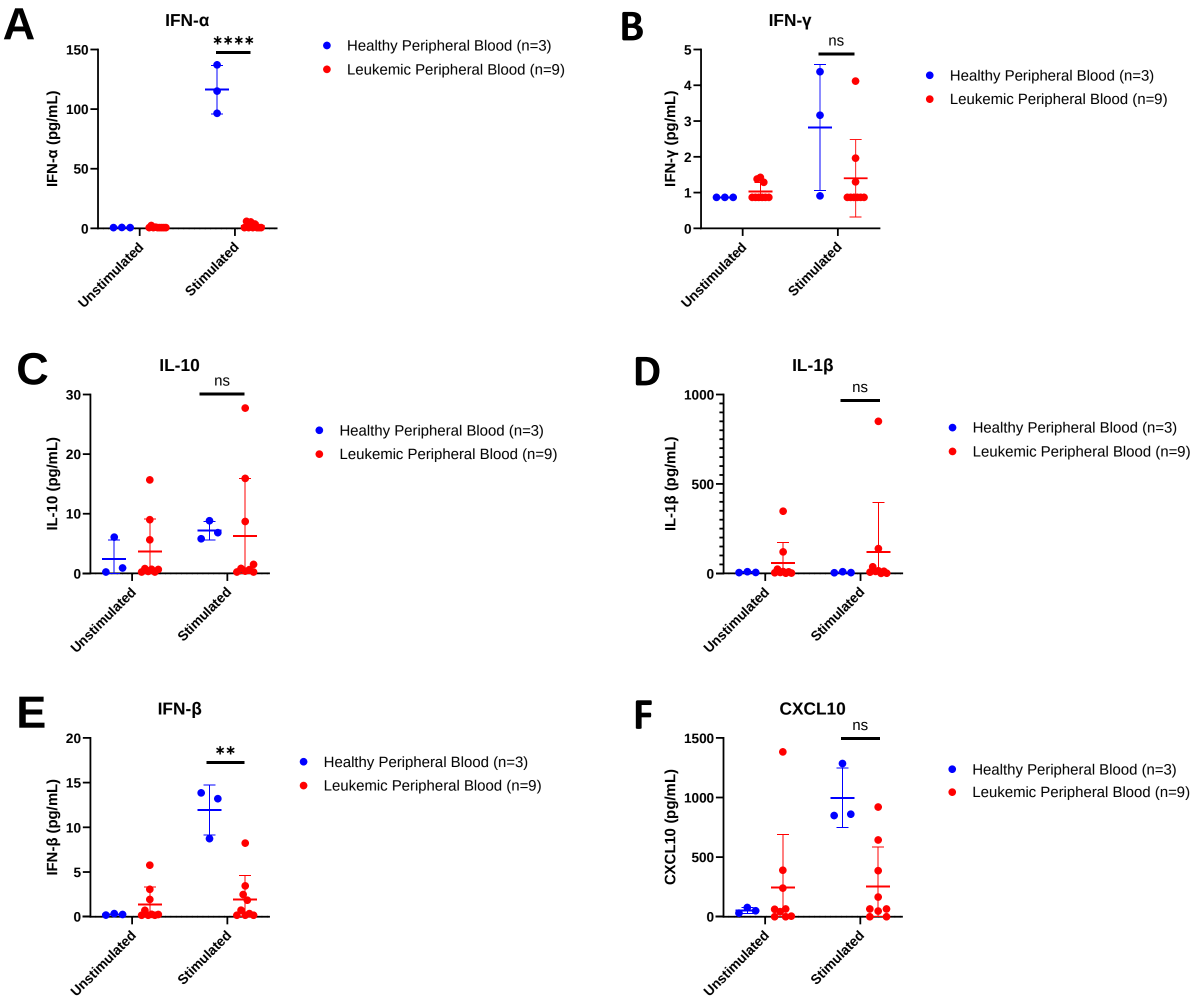

Supplemental Figure 4. Cytokine production from the supernatant of healthy and leukemic peripheral blood cells after overnight stimulation with 10µg of CpG. Concentration of (**A**) IFN-α, (**B**) IFN-γ, (**C**) IL-10, (**D**) IL-1β, (**E**) IFN-β, and (**F**) CXCL10 in pg/mL was measured. Data are presented as mean ± standard deviation. **(FDR)$P \leq 0.01$ and ****(FDR)$P \leq 0.0001$ from ANOVA.

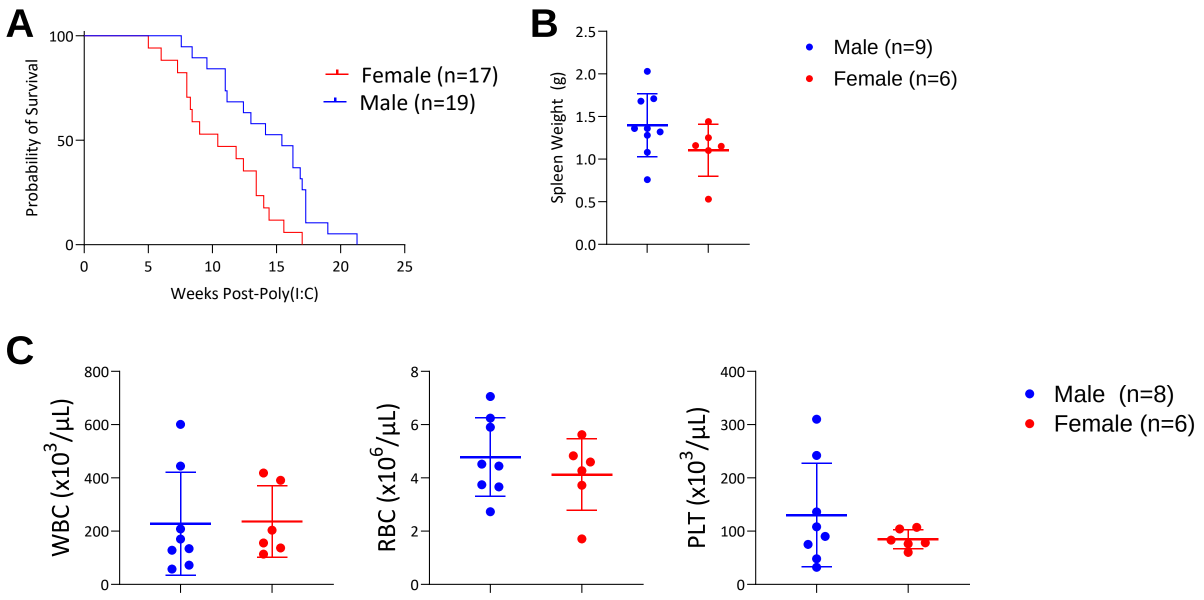

Supplemental Figure 5. Sex differences in the novel *Npm1*^{cA}/*Ptpn11*^{E76K} mouse. (**A**) Overall survival of *Npm1*^{cA}/*Ptpn11*^{E76K} mice based on sex. (**B**) Spleen weight in grams (g) of *Npm1*^{cA}/*Ptpn11*^{E76K} mice at death based on sex. (**C**) White blood cell count (WBC), red blood cell count (RBC), and platelet (PLT) count of *Npm1*^{cA}/*Ptpn11*^{E76K} mice at death based on sex. Data are presented as mean ± standard deviation.

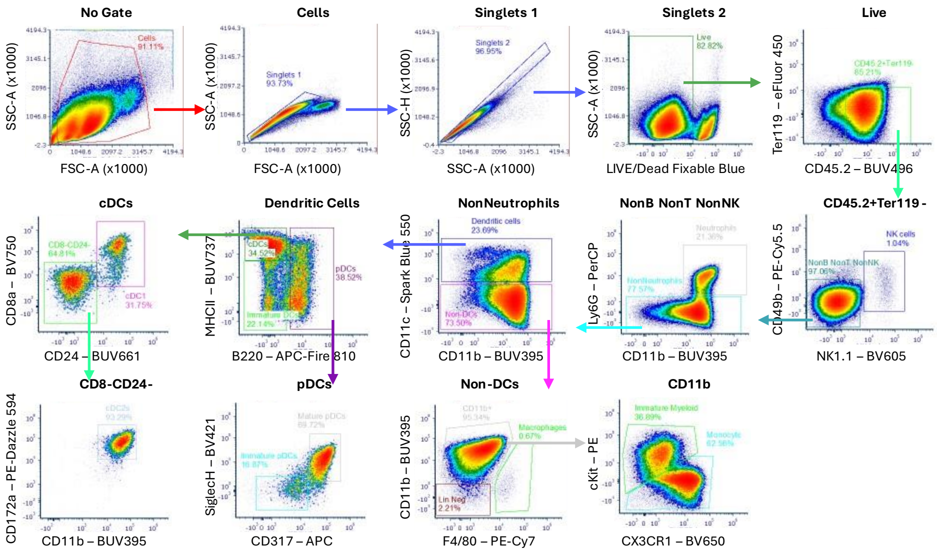

Supplemental Figure 6. Gating strategy for spleen immunophenotyping panel for leukocyte populations.

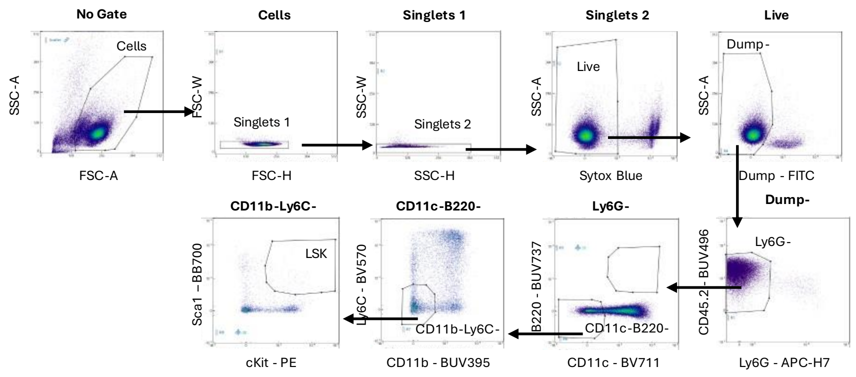

Supplemental Figure 7. Sorting strategy for LSK engraftment experiments. Dump channel includes CD3, Ter119, and FcεR1.

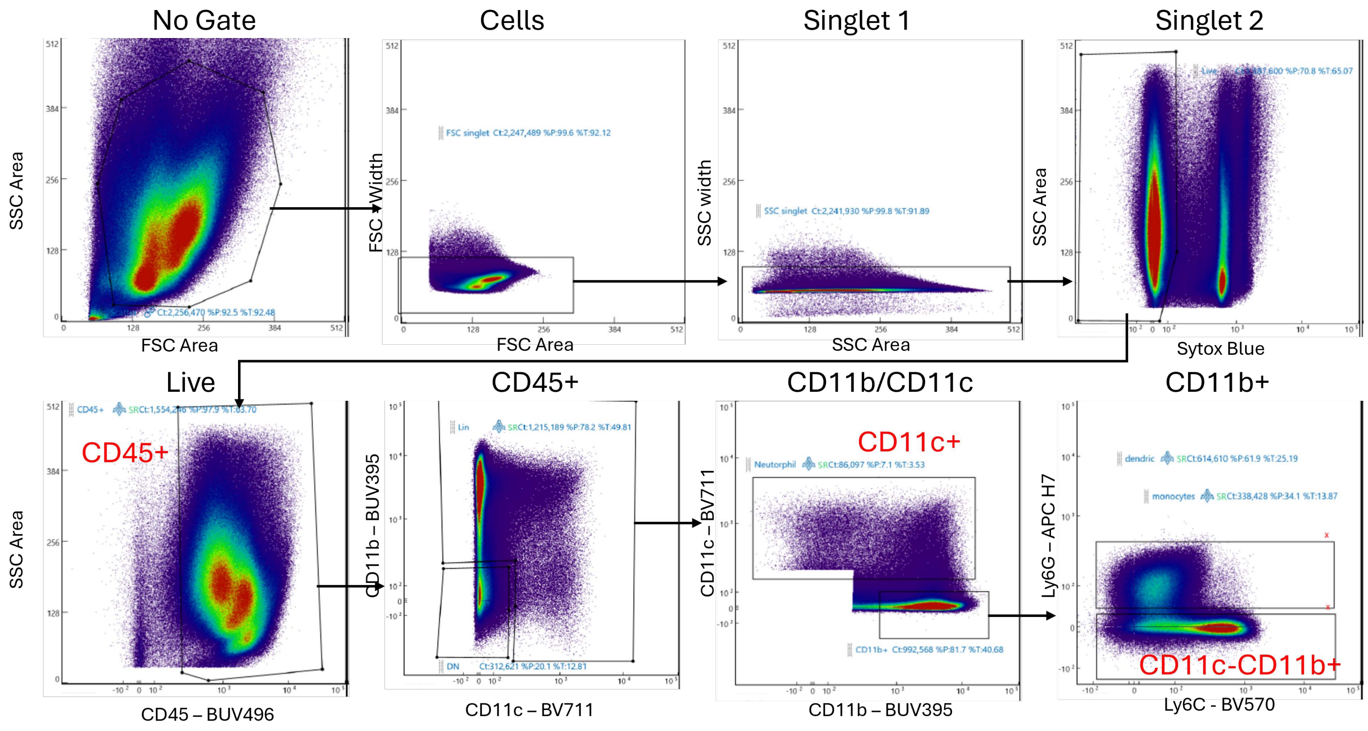

Supplemental Figure 8. Sorting strategy for lineage positive population engraftment experiments.
